# Respiratory syncytial viral load drives ciliated cell dedifferentiation and suppresses antiviral immunity

**DOI:** 10.1126/sciadv.aed4499

**Published:** 2026-06-19

**Authors:** Kevin Berg, Sibylle Haid, Ehsan Vafadarnejad, Arnaud Carpentier, Robert Geffers, Bettina Wiegmann, Antoine-Emmanuel Saliba, Florian Erhard, Thomas Pietschmann

**Affiliations:** ^1^ Institute of Virology and Immunology, Julius-Maximilians-University Würzburg, Würzburg, Germany.; ^2^ Faculty for Informatics and Data Science, University of Regensburg, Regensburg, Germany.; ^3^ Institute for Experimental Virology, TWINCORE - Centre for Experimental and Clinical Infection Research, a Joint Venture between Helmholtz Centre for Infection Research and the Hannover Medical School, Hannover, Germany.; ^4^ Biomedical Research in Endstage and Obstructive Lung Disease Hannover, (BREATH), German Center for Lung Research (DZL), Germany.; ^5^ Helmholtz Institute for RNA-based Infection Research (HIRI), Helmholtz-Center for Infection Research (HZI), Würzburg, Germany.; ^6^ Genome Analytics, HZI, Braunschweig, Germany.; ^7^ Department of Cardiothoracic, Transplantation and Vascular Surgery, Hannover Medical School, Hannover, Germany.; ^8^ Lower Saxony Center for Biomedical Engineering, Implant Research and Development (NIFE), Hannover, Germany.; ^9^ Institute of Molecular Infection Biology Faculty of Medicine, University of Würzburg, Würzburg, Germany.; ^10^ Cluster for Nucleic Acid Sciences and Technologies – NUCLEATE, Würzburg, Germany.; ^11^ German Center for Infection Research (DZIF), Site Hannover-Braunschweig, Germany.

## Abstract

Respiratory syncytial virus (RSV) causes severe lower respiratory disease, yet how it reshapes airway epithelial cells and evades innate immunity remains incompletely understood. We infected adult primary human airway epithelial cultures with RSV and analyzed infected and bystander cells over time using single-cell RNA sequencing and imaging. RSV mainly infected ciliated cells, triggering a virus load–dependent shutdown of genes involved in ciliogenesis, antigen presentation, and innate sensing, including key interferon (IFN) and pattern recognition pathways. Only a subset of infected cells produced type I and III IFNs, while bystander cells exhibited strong IFN-stimulated gene (ISG) signatures. Neither IFN treatment nor ISG induction eliminated infection, but IRF1, an antiviral transcription factor not suppressed by RSV, remained robustly expressed. Ectopic IRF1 expression in vitro reduced viral replication. These findings reveal how RSV evades antiviral defenses and highlight IRF1 as a potential target for therapeutic intervention.

## Teaser

RSV reshapes airway cells and escapes and weakens immune control through viral load–dependent transcriptional reprogramming.

## INTRODUCTION

Respiratory syncytial virus (RSV) is a globally prevalent respiratory pathogen that causes seasonal epidemics across all age groups, with the highest disease burden in infants, older adults, and individuals with chronic or immunocompromising conditions (*1*, *2*). While RSV infection is typically mild and self-limiting in healthy adults, severe lower respiratory tract disease can occur in vulnerable populations, leading to pneumonia, respiratory failure, hospitalization, and increased mortality (*2*, *3*). Preventive options have improved with the recent approval of vaccines and monoclonal antibodies, yet their efficacy remains incomplete, and therapeutic options for established RSV infection are limited (*4*–*6*). Identifying host processes that govern RSV replication and pathogenesis therefore remains a critical goal.

RSV-associated lung disease is likely driven in part by direct effects of viral infection on the respiratory epithelium. Evidence from human pathology, ex vivo tissue analysis, and differentiated primary airway epithelial cultures indicates that RSV exhibits a strong tropism for apical ciliated epithelial cells of the conducting airways (*7*–*11*). In fatal infant cases, viral antigen is prominent in bronchiolar epithelium, and infection of type I and type II pneumocytes has also been documented, indicating that RSV tropism is concentrated in, but not strictly limited to, ciliated airway cells (*9*). This preferential targeting of ciliated cells is recapitulated in well-differentiated primary human airway epithelial cultures, including pediatric models, which reproduce key hallmarks of infection observed in vivo (*7*–*11*). Infection of nonciliated epithelial populations appears to be more context dependent. In particular, basal cells are largely protected in intact pseudostratified epithelium but can become infected after epithelial injury or when basolateral access is exposed, and in this setting, RSV infection can alter epithelial repair and differentiation through interferon (IFN)–dependent mechanisms that favor secretory over ciliated cell fate (*10*). In parallel, the RSV infection of differentiated pediatric airway epithelium is associated with ciliated-cell damage, cell rounding, shedding, and occasional syncytium formation, supporting the concept that epithelial injury is a direct contributor to disease pathogenesis (*8*). Recent work has strengthened this model by showing that infant airway epithelium is especially susceptible to RSV-induced apoptotic extrusion of infected ciliated cells, impaired epithelial survival signaling, enhanced viral spread, and heightened inflammatory injury, whereas adult airway epithelium is relatively more resistant to this cytopathic response (*11*). Together, these findings support a model in which preferential infection of ciliated epithelial cells, followed by epithelial apoptosis, sloughing, and dysregulated repair, contributes directly to bronchiolar obstruction and lung disease severity during RSV infection (*7*–*11*). However, how RSV alters epithelial cell identity and differentiation state during the course of infection remains incompletely understood.

RSV is detected by multiple innate immune sensing pathways, but the resulting antiviral response is both incomplete and actively counteracted by the virus (*12*–*14*). In infected airway epithelial cells, the cytosolic recognition of RSV RNA by RIG-I, with contribution from MDA5 in some settings, activates MAVS-dependent signaling and induces type I and type III IFNs together with inflammatory mediators such as interleukin-6 (IL-6), IL-8, and other chemokines (*12*–*17*). Additional sensing through Toll-like receptors—including Toll-like receptor 3 (TLR3), TLR4, and TLR7 (*18*–*20*)—has been described in epithelial and myeloid cells and contributes to nuclear factor κB– and interferon regulatory factor (IRF)–dependent inflammatory and antiviral programs (*21*–*23*). Not all innate immune activation is equivalent. The experimental activation of RIG-I can induce a cell-intrinsic antiviral state that limits RSV replication (*24*), whereas a human challenge study suggests that a preexisting inflammatory mucosal milieu may increase susceptibility to infection (*25*). These observations can be reconciled by distinguishing protective antiviral IFN programs from baseline inflammatory states that reflect epithelial stress or dysregulated immune activation but do not efficiently restrict viral entry and replication. Consistent with this, the human challenge study (*25*) and pediatric transcriptomic analyses (*26*) indicate that the preexisting and early induced mucosal innate immune state is a major determinant of susceptibility, viral control, and disease severity. RSV has evolved potent mechanisms to blunt these responses. The nonstructural proteins NS1 and NS2 are the principal viral antagonists of innate immunity and suppress both IFN induction and downstream signaling through several mechanisms (*27*–*29*), including interference with RIG-I/MAVS–IRF3 signaling (*30*, *31*) and degradation or functional inhibition of signal transducers and activators of transcription 2 (STAT2) (*27*, *32*, *33*). In parallel, the RSV G glycoprotein functions as an immunomodulator in addition to its role in attachment, partly through its CX3C motif and interactions with CX3CR1-linked pathways, thereby altering leukocyte recruitment and host inflammatory responses (*34*–*36*). Together, these findings support a model in which RSV replication, epithelial inflammation, and tissue injury reflect the balance between early innate sensing and the virus’s ability to suppress or divert IFN-driven antiviral defenses.

Here, we investigated RSV-induced epithelial and immune responses using a primary human pseudo-stratified airway epithelial cell model that recapitulates key features of the human airway epithelium (*8*). By combining time-resolved single-cell RNA sequencing with imaging and functional assays, we analyzed infected and bystander cells across a wide range of viral RNA loads. This approach allowed us to reconstruct continuous host transcriptional changes along the RSV infection cycle at single-cell resolution. We show that increasing RSV viral load drives progressive dedifferentiation of infected ciliated cells toward a basal-like state, suppresses antigen presentation and IFN responses, and induces cellular stress pathways linked to apoptosis. In contrast, selected IFN-stimulated regulators, including IRF1, escape viral suppression and restrict RSV replication when ectopically expressed. Together, these findings reveal how RSV dynamically reprograms airway epithelial cells to evade innate immunity and identify IRF1 as a potential target to reinforce epithelial antiviral defense.

## RESULTS

### RSV infection produces distinct transcriptional states in infected and bystander airway cells

**Fig. 1.**
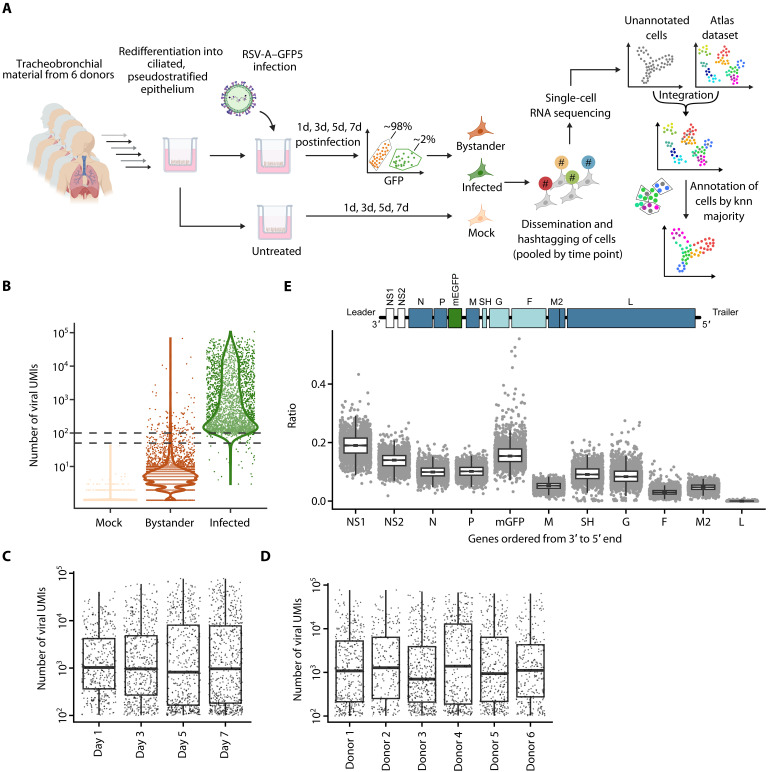
Study design and viral gene expression of RSV infected cells. (**A**) Tracheobronchial samples from six donors were cultured in ALI and infected with a GFP-expressing RSV-A strain. The cells were collected and fluorescence-sorted at days 1, 3, 5, and 7 postinfection, subsequently hashtagged with antibodies and analyzed by scRNA-seq. Cell-type annotation was done by the integration of the dataset with an atlas dataset, calculation of the nearest neighborhood graph, and annotation by majority of cell types in a cell’s neighborhood (see Materials and Methods and Figs. 2 and 3). Created in BioRender, S. Haid (2026); https://BioRender.com/pzx5jhl. (**B**) Violin plot showing the number of viral UMIs per cell. The dashed lines indicate the upper filter for bystander cells at 50 UMIs and the lower filter for infected cells at 100 UMIs. (**C**) Box plots of the viral UMIs at 1, 3, 5, and 7 days post infection. (**D**) Box plots of the viral UMIs across six donors. (**E**) Transcriptional profile of viral mRNA after manual RSV reannotation. The viral genome is given at the top, and the position of the GFP reporter gene is annotated.

To define how RSV infection shapes host transcriptional responses at single-cell resolution, we infected primary human airway epithelial cultures from six adult donors with a green fluorescent protein (GFP)–expressing RSV-A strain long reporter virus and analyzed cells at days 1, 3, 5, and 7 postinfection (Fig. 1A). Before single-cell RNA sequencing, cells were separated by fluorescence-activated cell sorting into RSV-infected (GFP-positive) and bystander (GFP-negative) populations, allowing enrichment of the otherwise rare infected cells (0.7 to 3.2% of total cells; fig. S1B). Nonsorted, mock-treated cultures from the same donors served as controls.

Single-cell RNA sequencing yielded 12,331 high-quality transcriptomes, including mock (*n* = 6496), infected (*n* = 2037), and bystander (*n* = 3798) cells, with an average of ~5700 detected genes per cell (fig. S2, A to D). Viral RNA abundance varied by more than three orders of magnitude among GFP-positive cells, ranging from fewer than 100 to more than 10,000 viral unique molecular identifiers (UMIs) per cell (Fig. 1B). In contrast, the vast majority of bystander cells contained fewer than 10 viral UMIs, consistent with minimal viral transcription or extracellular RNA contamination (Fig. 1B). To ensure robust separation of transcriptional states, subsequent analyses focused on infected cells with >100 viral UMIs and bystander cells with <50 viral UMIs.

Across all time points and all six donors, the distribution of viral RNA loads among infected cells was remarkably similar, with median viral counts of ~1000 UMIs per cell and a dynamic range spanning nearly three orders of magnitude (Fig. 1, C and D, and fig. S3). Because viral RNA levels are expected to increase over the course of infection within individual cells, we interpreted viral RNA abundance as a proxy for progression through the RSV replication cycle. The presence of cells with a high viral load on day one after inoculation and with low viral RNA loads at later time points suggests that the completion of the RSV replication cycle takes less than 24 hours in this model and that continuous rounds of infection and reinfection throughout the 7-day experiment occur. This interpretation is also supported by our observation that addition of palivizumab, a fusion protein targeting neutralizing antibody, 24 hours after RSV inoculation strongly reduced infected cell numbers 96 hours later (fig. S4).

Consistent with previous reports (*37*–*39*), viral mRNA expression decreased from the 3′ to the 5′ end of the viral genome, with the most 3′ transcription unit encoding the NS1 protein being the most abundant and the most 5′-terminal message of the L protein being the least transcribed mRNA (Fig. 1E). In our initial analysis of this gradient, the G protein mRNA was overrepresented relative to its position within the transcriptional gradient (fig. S5A). On closer inspection of the underlying read mapping pattern to the RSV reference genome (*40*), we found two irregularities. First, for four genes (mGFP, M, SH, and F), reads were not fully covered by the reference annotation, owing to a lacking annotation of 3′ untranslated regions (3′UTRs) in the reference (fig. S5B). Second and unique to the G protein sequence, we observed a second peak of mapped reads in the center of the gene body (fig. S5B), which substantially increased the overall G mRNA read counts. Examining the G gene sequence, we found several polyadenylate [poly(A)] stretches downstream of the second peak within the G protein coding sequence, similar in distance to the poly(A) signal and the primary peak (fig. S5C). During reverse transcription, these additional poly(A) sites could provide additional anchor points for poly(T) primer binding, resulting in an overrepresentation of the G protein mRNAs. Taken together, both the shortened 3′UTRs and the internal peak in the G protein message led to biased read counts for viral mRNAs. To address both irregularities, we instead created a manual, data-driven annotation of the RSV genome for the analysis of the transcriptional gradient using fixed 200–base pair (bp) read count analysis windows centered on the 3′ peaks for each gene. Using this curated annotation (fig. S5D), still an imperfect 5′ to 3′-polar mRNA gradient is apparent. The overrepresentation of the mGFP, SH, G, and M2 messages relative to their respective genome position (Fig. 1E) suggests distinct posttranscriptional regulation of these mRNAs.

### Mock gene expression profiles reflect the major epithelial cell types but infection globally perturbs gene expression

**Fig. 2.**
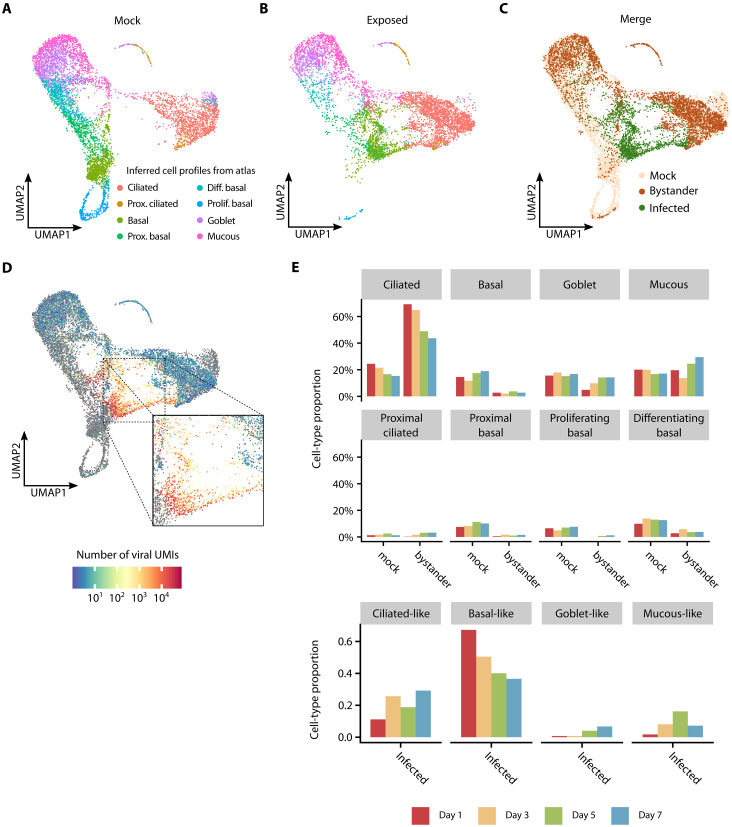
Cell profile annotation via human lung cell atlas. (**A**) UMAP representation of mock cell profiles annotated by integration with human lung cell atlas. (**B**) UMAP representation of RSV exposed cell profiles annotated by integration with human lung cell atlas. (**C**) UMAP representation of infection states of mock and exposed cells. (**D**) UMAP representation of the number of viral UMIs across infected cells. The inlay displays a magnified view of highly infected cells. (**E**) Bar plots of the relative proportion of cell types within mock, bystander, and infected cells across time points. The *x* axis represents the different infection states, and the *y* axis indicates the relative proportion of the cell type per time point and infection state, with the total proportions of all cell types per infection state and at each time point summing to 100%.

To investigate RSV infection at the single-cell level, we transferred cell-type annotations from a published human lung cell atlas (*41*) first to our mock cells (Fig. 2A). Label transfer was favored over marker gene based manual annotation of cell types to make use of the comprehensive lung cell atlas that has been annotated by large gene panels (see Materials and Methods). Ionocytes and Club cells were filtered out because only a handful of them could be captured (<10 cells). We identified a distinct cluster of 198 cells enriched with low quality that was also filtered out (fig. S2, B and C). Furthermore, we were able to assign basal and ciliated cell subpopulations (e.g., proliferating basal cells) identical to those previously assigned by the human lung cell atlas. This cell-type assignment was overall conserved across all six donors (fig. S2F). Thus, our culture model faithfully recapitulated the typical cell types of a pseudo-stratified human bronchial epithelial cell layer (*41*).

Next, using canonical correlation analysis, we mapped RSV exposed cells including bystanders and infected cells into a joint embedding with the mock cells for an automatic cell-type annotation of the exposed cells (Fig. 2, B and C). Overall, exposed cells matched well to mock cells with two exceptions (Fig. 2, A to C): First, all basal cell types (basal and proximal, proliferating, and differentiating basal) were underrepresented among exposed and absent from infected cells, presumably because the differentiation of basal cells is induced upon depletion of cells by infection; second, the majority of infected cells segregated away from all mock cell subsets and shared expression patterns of both ciliated and basal mock cells in a viral RNA load dependent manner. Infected cells with low viral RNA load appeared most similar to ciliated cells and were thus called “ciliated-like” (Fig. 2D). By contrast the infected cells with highest viral RNA load were rather correlated with basal cells and therefore called “basal-like”. This phenomenon was consistently observed throughout the days 1 to 7 investigated here and for all six donors (fig. S3).

To investigate these phenomena further, we examined the annotations of transferred cells and compared the quantitative changes in cell populations over time in mock-treated and bystander cells. Notably, the single-cell sequencing was conducted after fluorescence-activated cell sorting (FACS) sorting for bystander cells, but not for mock-treated cells. Therefore, we cannot rule out the possibility that this additional step has influenced the relative numbers of cell types among mock versus among bystander cells, for example, due to the cells having different capacities to survive the FACS sorting step. In mock-treated control cells, the relative numbers of different cell types remained fairly constant over days 1 to 7 with only a slight decrease in ciliated cells and a slight increase of basal cells over time, indicating that the cell culture is in a steady-state equilibrium when unperturbed (Fig. 2E). In contrast, the distribution of cell types was altered in the RSV-exposed bystander cell populations, with a pronounced reduction of proliferating basal cells, of proximal and differentiating basal cells, an overrepresentation of ciliated (or ciliated-like cells), and a time-dependent modulation of the ciliated, goblet, and mucous cell compartments. Ciliated cells initially increased and returned to almost baseline levels by day 7. Goblet cells initially decreased and returned to levels similar to those of mock-treated controls by day 7, while mucous cells increased on days 5 and 7 (see Fig. 2E). To complement these transcriptome data, we infected cells from three independent donors with RSV-A–GFP and monitored RSV propagation and cell types by flow cytometry using cell type–specific marker protein staining. In this independent experiment, RSV infected between 0.1 and 1.2% of total cells and maintained this infection rates with some fluctuation throughout the 7-day follow up (fig. S6D). When assessing time-dependent changes of ciliated cells using ARL13B and β-tubulin marker gene staining, we did not observe significant changes in ciliated cell numbers over time or between mock and bystander cells (fig. S6F). Similarly, when staining with CD49f or CD271 as basal cell marker proteins, no gross changes in basal cell populations were observed between mock and bystander cells over time (fig. S6G). In contrast, mucin 5 AC staining suggested a modest increase in goblet and mucous producing cells after exposure to RSV (fig. S6E). Together, the transcriptional profiling results showed that RSV infection caused an expansion of cells with a ciliated cell transcriptional profile, without depleting ciliated cells or spreading completely to all available ciliated cells. In contrast, exposure to RSV depleted cells with basal cell transcriptional profiles relative to their abundance in mock-treated controls. Notably, flow cytometry staining for selected cell lineage markers did not recapitulate these changes, possibly due to the focus on selected protein markers and the turnover rates of these proteins.

### RSV primarily infects ciliated cells and causes cell dedifferentiation

Previous studies in similar culture models have reported that RSV primarily targets ciliated cells (*7*, *8*, *10*, *42*, *43*). Likewise, histological analyses of RSV-infected pediatric lungs point toward ciliated cells as primary RSV target cells (*9*, *44*). It was therefore unexpected that the majority of RSV-infected cells with high viral RNA load had gene expression profiles reminiscent of basal cells and were distinct from ciliated cells (Fig. 2, A to D).

**Fig. 3.**
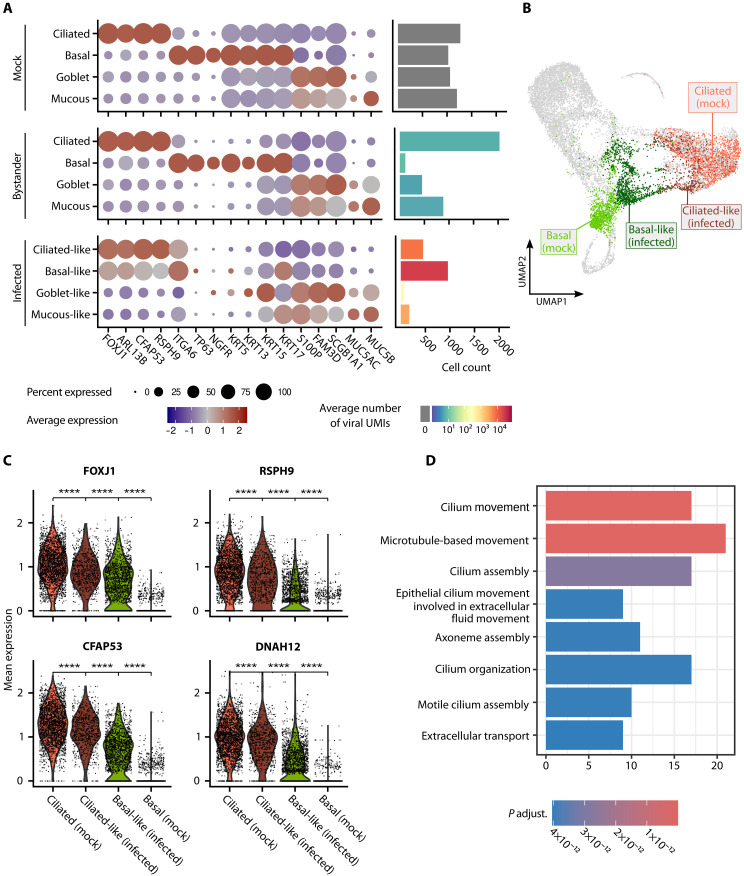
Infected ciliated cells with high virus load exhibit basal cell-like expression profile. (**A**) Average expression and percentage of expressing population of marker genes in mock, bystander, and infected ciliated, basal, goblet, and mucous cells (left). Cell counts per cell type and infection state, and the color represents the average natural log value of viral reads (right). (**B**) UMAP visualization of basal(-like) and ciliated(-like) cells. (**C**) Gene expression of four marker genes for ciliated cells and microtubules over ciliated, ciliated-like, basal-like, and basal cells. Wilcoxon test, *P* values indicated. (**D**) GO analysis of down-regulated genes in infected basal-like cells versus mock ciliated cells. The bars represent the gene count per category, and the color represents the respective adjusted *P* value. The top eight hits are shown.

To investigate the alterations in the gene expression profiles of infected cells, we first dissected cell type–specific marker gene expression in the mock, bystander, and infected cell populations (Fig. 3A and fig. S7). Mock cells comprised well-distinguishable cellular mRNA expression signatures separating ciliated and basal cells from each other. Very high expression of *FOXJ1*, *ARL13B*, *CFAP53*, and *RSPH9* characterized essentially all ciliated cells. In contrast, the vast majority of basal cells abundantly expressed *ITGA6*, *TP63*, *NGFR*, *KRT5*, *KRT13*, *KRT15*, and *KRT17* mRNAs (Fig. 3A, top). With the exception of *KRT5* and *KRT13*, all markers showed virtually the same expression patterns in the respective bystander cells.

By contrast, infected cells showed a strongly perturbed pattern of marker gene expression: In the ciliated-like cells, which were characterized by low viral RNA load, the expression of *FOXJ1*, *ARL13B*, *CFAP53*, and *RSPH9* was slightly reduced compared to mock ciliated cells. These ciliated cell marker genes were expressed even more weakly in the highly infected basal-like cells. This indicated that RSV reduced the expression of these genes over the course of its infection cycle. In contrast, the basal cell marker gene *ITGA6* was more strongly expressed in ciliated-like infected cells than in mock ciliated cells and showed even stronger expression in basal-like infected cells.

We also detected a few goblet-like and mucous-like infected cells with very low or low virus load, respectively. Again, their transcriptional profile was skewed compared to the respective cell populations in the mock-treated culture. These latter changes are most prominently represented by the up-regulation of *KRT15* in the goblet-like infected cells compared to the goblet cells and the up-regulation of *KRT17* in the mucous-like cells compared to the mucous cells. Collectively, these results indicate that RSV infection induces a profound transcriptional reprogramming that affects cell annotation and likely cell physiology. Using viral RNA load as a marker of the infection time of an individual cell, we hypothesized that ciliated cells were infected throughout the 7 days studied here and changed their gene expression profile toward a more basal cell-like profile as its infection cycle progressed, suggesting that rising virus RNA load may drive dedifferentiation of ciliated cells toward a basal-like phenotype.

To investigate this dedifferentiation in more detail, we compared the infected and mock cells annotated as “ciliated” or “basal” at the single-cell level (Fig. 3B). All four ciliated marker genes *FOXJ1*, *ARL13B*, *CFAP53*, and *RSPH9* were most strongly expressed in ciliated mock cells (Fig. 3C). In infected ciliated-like cells with low viral RNA load, ciliated marker gene expression was markedly reduced. This reduction was even stronger in infected basal-like cells with higher viral RNA load. The Gene Ontology (GO) analysis of the cumulated transcriptional changes distinguishing the infected basal-like cells from the ciliated mock cells identified a strong and significant underrepresentation of GOs involved in cilium assembly, organization, and movement but no other pathway that is unrelated to cilia (Fig. 3D). These observations are consistent with a model where ciliated cells are infected by RSV, which then lose their ciliated phenotype during the infection cycle.

**Fig. 4.**
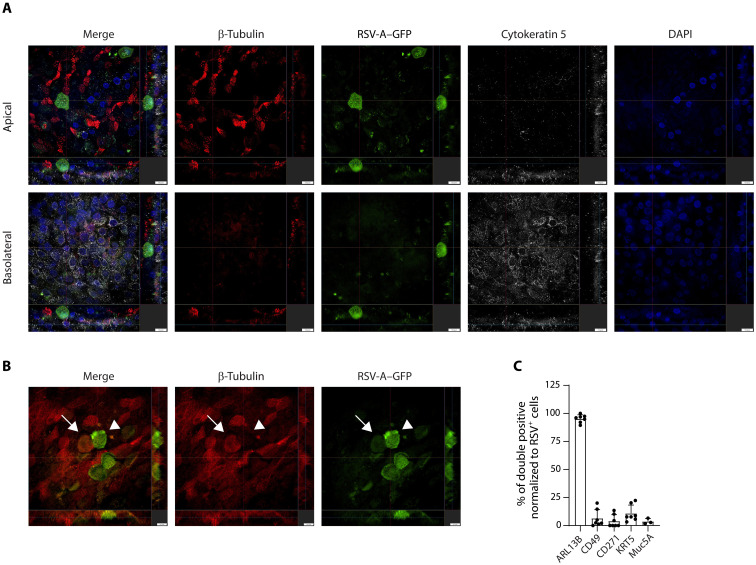
RSV primarily infects ciliated cells. (**A**) Immunofluorescence analysis of well-differentiated primary human airway epithelia cells cultured under ALI conditions and infected with an RSV-A–GFP reporter virus. β-tubulin primarily stains the cilia, whereas cytokeratin 5/KRT5 is a marker for basal cells. Two different projections (top row: apical; bottom row: basolateral) and z-stacks are displayed. Scale bar, 100 μm. One representative set of pictures is given. (**B**) Immunofluorescence analysis of primary human airway epithelial cells infected with RSV-A–GFP reporter virus. β-tubulin staining is reduced in cells with high GFP expression (compare β-tubulin staining in cells with weak GFP expression (arrow) with cells with strong GFP expression (arrow head). (**C**) Flow cytometric analysis of well-differentiated human airway epithelia cells. Percentage of RSV-infected cells positive for respective marker protein is depicted. Symbols represent individual donors from *n* = 2 independent experiments. DAPI, 4′,6-diamidino-2-phenylindole.

To directly confirm ciliated cells as the primary target cells of RSV infection, we performed immunofluorescence microscopy and flow cytometry using markers of ciliated and basal cells (Fig. 4). RSV-infected cells were predominantly localized to the apical surface of the epithelium and coexpressed the ciliated cell marker ARL13B or β-tubulin respectively, whereas only a small fraction expressed the basal cell marker KRT5 (Fig. 4, A to C). Moreover, the β-tubulin staining of cilia was markedly reduced in cells with high GFP expression compared with weakly infected cells (Fig. 4B), consistent with virus load–dependent loss of ciliary structures.

Together, these data demonstrate that RSV primarily initiates infection in ciliated airway epithelial cells and drives a viral load–dependent dedifferentiation program that progressively suppresses ciliogenesis and induces a basal-like transcriptional state.

### RSV viral load progressively suppresses IFN signaling in infected cells

**Fig. 5.**
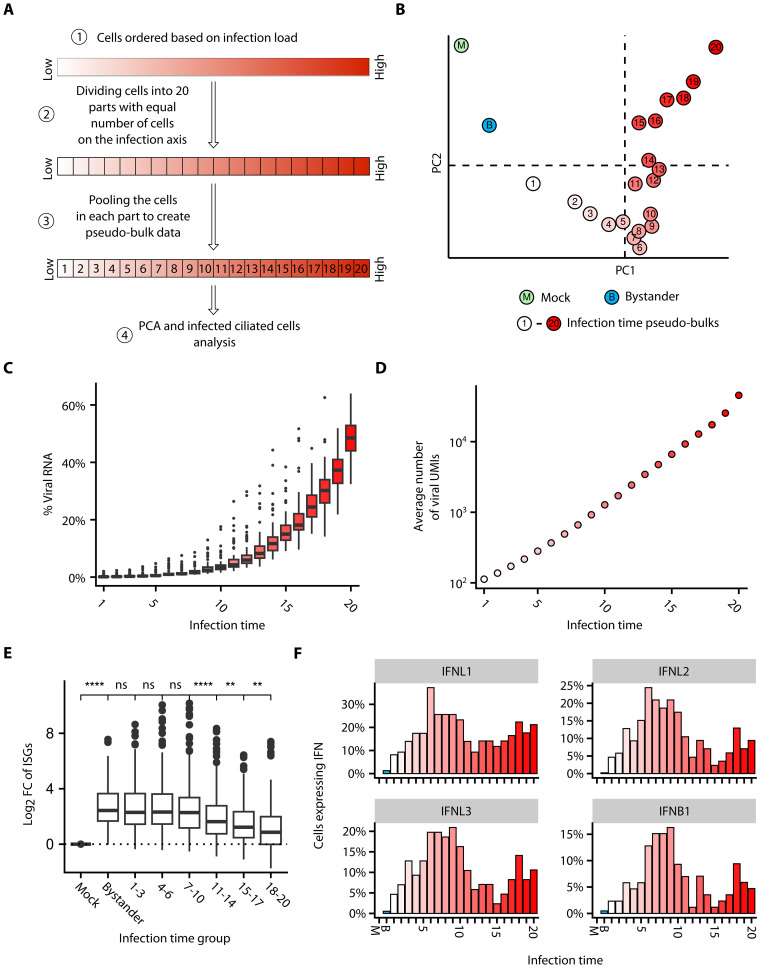
Pseudobulk analysis reveals infection time-dependent blunting of the interferon response. (**A**) Scheme of the pseudobulk analysis to investigate viral load–dependent transcriptional regulation. Cells are equally divided along the viral load axis into 20 bins, which are then subjected to principal components analysis (PCA) and differential gene expression analyses. (**B**) PCA plot of the first two principal components of the binned reads for mock (green), bystander (blue) and 20 viral load bins (red gradient). (**C**) Boxplots showing the percentage of viral RNA for all infected cells per infection time pseudobulk. (**D**) Average number of viral UMIs per infection time pseudobulk. (**E**) Visualization of the interferon score (mean log_2_FC of IFN hallmark genes) across mock and bystander cells and grouped viral load bin. Wilcoxon test, *P* values indicated. (**F**) Percentage of cells expressing IFNL1-3 or IFNB1 over mock, bystander, and infection time pseudo bulks.

Because infected cells displayed similar transcriptional profiles across experimental time points (Fig. 1C), we pooled infected cells from all days and donors ordered them by viral RNA abundance as a proxy for progression through the RSV replication cycle. To analyze transcriptional changes along this continuum, we divided infected cells into 20 pseudobulk groups of equal size spanning low to high viral RNA loads (Fig. 5A and fig. S8). Principal components analysis revealed a gradual and continuous shift in host transcriptional states across these pseudobulks, distinct from both mock-treated ciliated cells and RSV-exposed bystander cells (Fig. 5B). Viral RNA constituted an increasing fraction of total cellular transcripts across pseudobulks, confirming progressive viral takeover at higher infection stages (Fig. 5, C and D).

RSV infection induces an IFN response in the human lung (*45*–*49*), in primary human cells (*50*, *51*), and in animal models (*52*), although IFN responses in RSV infection of human cells may be weaker than in influenza A virus infection (*45*, *50*). Such a comparatively weak IFN response to RSV may be due to the RSV proteins NS1 and NS2 blunting IFN induction and signaling (*12*, *53*). To quantify RSV-induced ISG induction, we thus examined a predefined set of typical ISGs and computed an ISG score reflecting the overall activation of the IFN pathway per cell. Comparing this ISG score among ciliated mock cell populations, all bystander cells and across the 20 infection time pseudobulks revealed a strong IFN response in bystander cells and a stable ISG expression throughout infection time 1 to 10 (Fig. 5E). Thereafter, the strength of the IFN response decreased rapidly and continuously at later infection time points. Thus, with increasing viral RNA load, IFN signaling was attenuated, probably due to NS1- and NS2-dependent interference with IFN signaling in the infected cells (*12*, *53*). Analyzing the IFN response over the 7 days of infection in bystander cells revealed a burst of ISG induction on day 1 and subsequent maintenance of a continuously high IFN score until day 7. Moreover, virus load classes above 10 exhibited a decreased ISG induction compared with lower virus load classes across all time points (fig. S9).

Consistent with this pattern, transcripts encoding type I and III IFNs (IFNB1 and IFNL1-3) were detected in only a minority of infected cells (~10 to 20%) at early infection stages and declined markedly at higher viral RNA loads (Fig. 5F). Interferon transcripts were largely absent from bystander cells, indicating that a small subset of infected cells is responsible for IFN production that drives widespread ISG induction in surrounding uninfected cells.

**Fig. 6.**
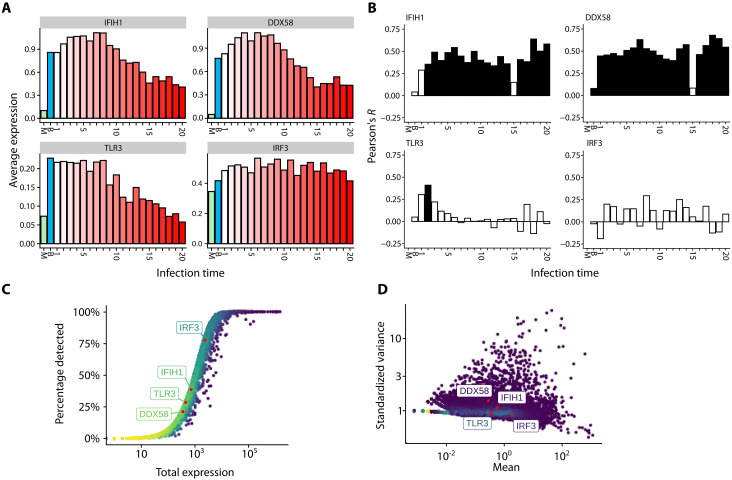
Regulation of PRR mRNAs in mock ciliated, bystander, and RSV infected cells. (**A**) Average mRNA expression of given PRRs and IRF3 in mock, bystander and infected cells. Expression in infected cells was quantified across the gradient of virus load. (**B**) Correlation analysis between mRNA expression of IFIH1 (MDA5), DDX58 (RIG-I), TLR3 (TLR-3), IRF3, and type I and III IFN expression among cells for each pseudobulk. Black bars indicate statistical significance (*P* < 0.05, *t* test, Bonferroni correction). (**C**) Relative number of mock treated cells (in %) expressing a given mRNA is displayed on the *y* axis and total average expression of the given mRNA on the *x* axis. (**D**) Variance of in total 24,800 mRNAs detected in the pool of mock-treated ciliated cells, standardized by the respective average expression value (standardized variance), is plotted relative to the mean total expression. The DDX58 mRNA ranks at 1034 and thus within the top 5% most variable mRNAs.

We next examined expression of pattern recognition receptors (PRRs) involved in RSV sensing. Transcripts encoding RIG-I (DDX58), MDA5 (IFIH1), and TLR3 were strongly induced in bystander cells and in infected cells with low viral RNA loads but decreased progressively at higher viral loads (Fig. 6A). In contrast, the expression of IRF3, a key transcription factor downstream of PRR signaling, remained stable or modestly increased across the viral load gradient (Fig. 6A). Correlation analysis revealed that IFIH1 and DDX58 expression positively correlated with IFN expression in early infection pseudobulks (*R* > 0.33, *P* < 0.05, *t* test, Bonferroni corrected), whereas this relationship was lost at later stages of infection (Fig. 6B). These findings are consistent with efficient viral suppression of PRR-dependent IFN induction as viral replication progresses.

Notably, the analysis of mock-treated ciliated cells revealed substantial cell-to-cell heterogeneity in basal PRR expression. Less than 25% of mock-ciliated cells expressed detectable DDX58 transcripts, and DDX58 ranked among the 5% most variable genes in this population (Fig. 6, C and D). This intrinsic heterogeneity suggests that only a subset of cells is poised to rapidly sense RSV infection and initiate IFN production, potentially explaining why IFN induction is confined to a minority of infected cells.

To assess whether enhancing IFN signaling could suppress RSV replication, we treated infected airway epithelial cultures with exogenous type I IFN-α. IFN-α treatment did not reduce the frequency of RSV-infected cells, whereas the pharmacological inhibition of STAT1 signaling with ruxolitinib markedly increased infection (fig. S4). These results indicate that while endogenous IFN signaling restricts RSV spread at the population level, RSV effectively neutralizes IFN-mediated antiviral defenses within infected cells once viral replication is established. Together, these data show that RSV elicits a transient IFN response early during infection that is progressively blunted as viral RNA accumulates, allowing infected cells to evade IFN production and innate immune control while bystander cells remain partially protected by sustained ISG expression.

### Viral load–dependent transcriptional reprogramming reveals cellular stress responses and identifies IRF1 as an unsuppressed antiviral regulator

**Fig. 7.**
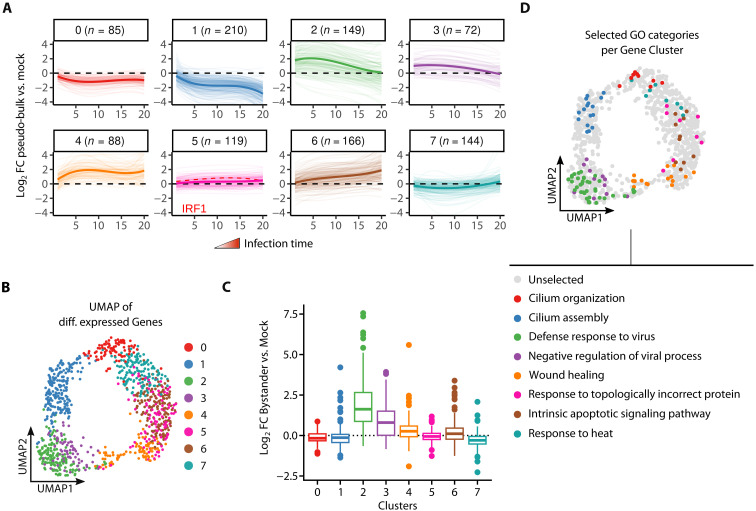
Uncovering viral load dependent transcriptional clusters by pseudobulk analysis. (**A**) Mean log_2_FC values versus mock per gene cluster and pseudobulk depict differing regulation patterns and magnitudes over infection time. All genes per cluster are shown as transparent lines, and the opaque line represents the regression line over all genes. IRF1 is highlighted as a dashed red line. (**B**) UMAP representation of gene clusters of all differentially expressed genes over the infection time based on their log_2_FC values in all pseudobulks. (**C**) Boxplots showing the log_2_FCs of gene expression in bystander versus mock cells over all clusters. (**D**) UMAP representation of gene clusters. Genes associated with representative GO terms are highlighted.

To comprehensively define host transcriptional programs associated with increasing RSV replication, we compared gene expression across the 20 viral RNA load pseudobulks to mock-treated ciliated cells. In total, 1033 genes were differentially expressed across the infection continuum (adjusted *P* < 0.01). Clustering these genes based on their expression dynamics revealed eight distinct temporal patterns of regulation (Fig. 7A and fig. S10).

To visualize similarities among these temporal expression profiles, we embedded genes into a two-dimensional space using uniform manifold approximation and projection (UMAP) based on their regulation across the viral load gradient. This gene-centric UMAP revealed a continuous circular organization, with the eight clusters occupying distinct but connected segments (Fig. 7B). This structure indicates that RSV-induced host transcriptional responses form a continuum rather than discrete states, with cluster centroids representing prototypical regulatory trajectories. Clusters 0 and 1, comprising ~29% of regulated genes, showed continuous or progressive down-regulation with increasing viral load and were strongly enriched for GO terms related to cilium assembly, organization, and motility (Fig. 7D and table S1). These findings further support virus load–dependent loss of ciliated cell identity. Notably, cluster 1 also included genes involved in major histocompatibility complex class II assembly, including *HLA-DPA1*, *HLA-DPB1*, and *HLA-DMA*, indicating the suppression of antigen presentation pathways during RSV infection (fig. S11).

Clusters 2 and 3 encompassed genes that were strongly induced at low viral RNA loads but declined as viral replication progressed (Fig. 7A). These clusters were enriched for IFN-stimulated genes and antiviral effectors, including *STAT1*, *CXCL10*, *TLR3*, and *IFITM1-3*, reflecting early activation and subsequent suppression of antiviral defense programs (Fig. 7D). In contrast, cluster 4 genes were rapidly induced early during infection and remained elevated across the viral load spectrum. These genes were associated with tissue remodeling and epithelial barrier maintenance, including *VEGFA*, *FGF2*, and *CLDN1*, suggesting an early injury response in infected cells.

Clusters 5 and 7 showed minimal regulation across infection stages and included genes involved in protein folding, cellular homeostasis, and stress adaptation (Fig. 7A). Intermixed within this region, cluster 6 genes displayed progressive up-regulation at higher viral loads and were enriched for pathways related to metabolic stress and apoptosis, including *DDIT4*, *CHAC1*, *IER3*, and *BID* (Fig. 7D). Consistent with these transcriptional changes, the pharmacological inhibition of apoptosis increased the number of detectable RSV-infected cells (fig. S4), indicating that cell death contributes to limiting infection at later stages. Together, these clusters depict a dynamic progression from immune activation to cellular exhaustion, loss of epithelial integrity, and ultimately, apoptosis, emphasizing RSV’s ability to drive airway dysfunction.

**Fig. 8.**
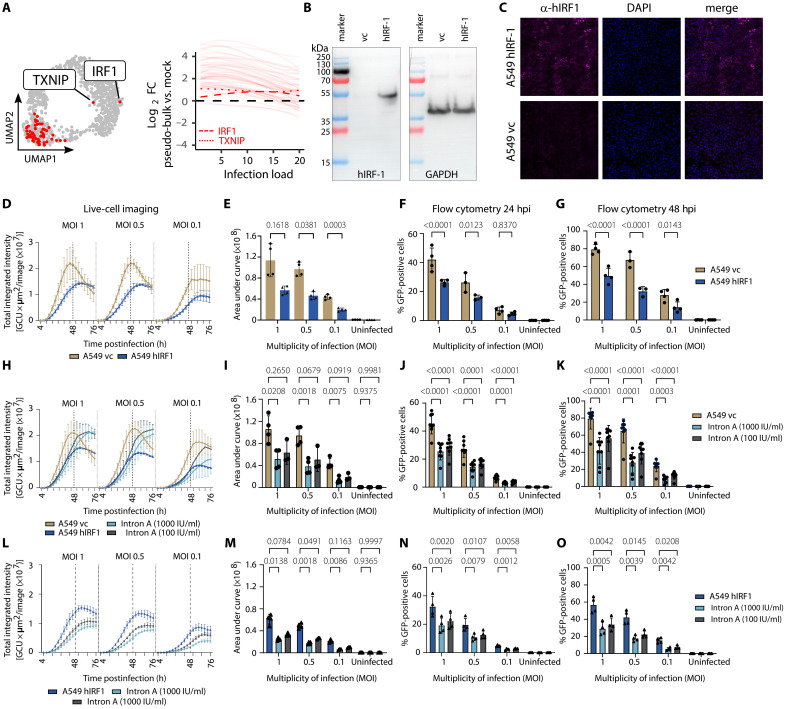
Influence of IRF1 overexpression on RSV infection. (**A**) UMAP representation of ISGs (red) and IRF1 and TXNIP. (**B**) Lysates of A549 cells either transduced with an empty vector control (vc) or an IRF1 expression construct (IRF-1) immunoblotted with IRF1- or GAPDH-specific antibodies. (**C**) Immunofluorescence analysis using an IRF1-specific antibody (magenta) and DAPI staining. (**D**) A549 vector control (brown) or A549 cells stably overexpressing IRF1 (blue) were inoculated with an RSV-A–GFP reporter virus at indicated multiplicities of infection (MOI), and GFP expression was quantified by live-cell imaging. (**E**) Area under the curve from (D) until the time point 48 hours post inoculation (hpi) was calculated and analyzed using a repeated-measurement one-way analysis of variance (ANOVA) in combination with Sídák’s multiple comparison test. Mean and SD of *n* = 4 independent experiments [(D) and (E)] as well as the results from the single experiments [symbols, (E)] are given. (**F** and **G**) Flow cytometric analysis at 24 hours (F) and 48 hours (G) postinfection. Bars represent mean and SD of *n* = 3 to 4 independent experiments. The results from each independent experiment are depicted as symbol. Two-way ANOVA with Sídák’s multiple comparison test. (**H** to **K**) RSV-GFP infection data of given cells pretreated with given quantities of interferon-2alpha (*n* = 3 to 4 independent experiments). [(H) and (I)] Live-cell imaging. For better comparison, the data from the A549 IRF1-overexpressing cells from [(D), blue line] was added to (H). [(J) and (K)] Flow cytometric analysis 24 or 48 hpi (*n* = 8 independent experiments). Bars represent mean and SD including the results for each independent experiment (symbol). Two-way ANOVA with Sídák’s multiple comparison test. (**L** to **O**) A549 IRF1 cells pretreated with interferon-2alpha and RSV infected. [(L) and (M)] live cell imaging analysis (*n* = 3 to 4 independent experiments); mixed-effects analysis and Sídáks multiple comparison test. [(N) and (O)] Flow cytometric analyses of cells 24 hpi (N) and 48 hpi (O) from *n* = 4 independent experiments. hIRF1, human IRF1.

Notably, not all IFN-stimulated genes followed the canonical induction-and-suppression pattern. Interferon regulatory factor 1 (*IRF1*) and thioredoxin-interacting protein (*TXNIP*) clustered separately (cluster 5) and maintained stable expression across the viral load gradient (Fig. 8A). To test whether this resistance to suppression translated into antiviral activity, we generated A549 cells stably overexpressing IRF1 or TXNIP (Fig. 8, B and C, and fig. S12M). IRF1 overexpression significantly reduced RSV replication across a range of viral inocula, as measured by live-cell imaging and flow cytometry (Fig. 8, D to G). In contrast, TXNIP overexpression had no detectable effect on viral replication (fig. S12, A to L).

We next compared the antiviral effect of IRF1 overexpression with that of IFN treatment. Pretreatment of control A549 cells with type I IFN-α delayed RSV replication but failed to prevent viral spread at later time points, particularly at higher multiplicities of infection (Fig. 8, H to K). In contrast, IRF1 overexpression resulted in sustained suppression of RSV replication that was not overcome by increased viral input (Fig. 8, D and E). Moreover, IFN-α treatment further enhanced antiviral protection in IRF1-overexpressing cells (Fig. 8, L to O), suggesting that IRF1 restricts RSV through mechanisms that are at least partially independent of canonical IFN signaling.

Together, these data demonstrate that RSV induces a coordinated, viral load–dependent transcriptional reprogramming of airway epithelial cells that integrates loss of epithelial identity, suppression of antiviral and antigen presentation pathways, induction of cellular stress responses, and eventual apoptosis. Within this landscape, IRF1 emerges as a host antiviral regulator that escapes viral suppression and effectively restricts RSV replication.

## DISCUSSION

RSV infection remains a major cause of lower respiratory tract disease, yet the cellular mechanisms by which the virus reshapes the airway epithelium and evades innate immunity have remained incompletely understood. Here, using time-resolved single-cell transcriptomics of primary human airway epithelial cultures, we show that RSV induces a continuous, viral load–dependent reprogramming of infected epithelial cells. This process is characterized by progressive loss of ciliated cell identity, suppression of antiviral and antigen presentation pathways, induction of cellular stress responses, and eventual apoptosis. At the same time, a small subset of infected cells initiates IFN production, which establishes a robust antiviral state in surrounding bystander cells and limits viral spread at the population level.

Our data confirm that ciliated cells are the primary targets of RSV infection in the human airway epithelium (*7*–*11*). We demonstrate that the basal-like transcriptional state observed in highly infected cells does not reflect preferential infection of basal cells but rather a virus-driven dedifferentiation of infected ciliated cells. This conclusion is supported by viral load–dependent erosion of ciliogenesis gene expression, selective down-regulation of cilia-associated pathways, and protein-level validation showing loss of ciliary markers in heavily infected cells (Figs. 3 and 4). These findings extend earlier observations of ciliated cell loss in RSV infection (*8*, *10*, *54*, *55*) by identifying dedifferentiation as a dynamic, infection-intrinsic process rather than a purely regenerative or injury-induced response.

A central finding of this study is the strong dependence of host antiviral responses on viral RNA load within individual cells. Early during infection, a subset of infected cells activates PRRs and induces type I and III IFNs, which in turn drive sustained IFN-stimulated gene expression in bystander cells (*13*, *14*, *27*–*29*, *45*–*51*, *56*, *57*). However, as viral replication progresses, infected cells progressively lose the ability to express IFNs and key upstream sensors, consistent with the activity of RSV immune antagonists such as NS1 and NS2 (*12*, *27*–*29*, *53*, *56*). This viral load–dependent silencing provides a unifying explanation for the paradoxical observation that RSV induces a strong IFN response at the tissue level while remaining refractory to IFN-mediated clearance at the level of infected cells.

The confinement of IFN production to a minority of infected cells likely reflects intrinsic heterogeneity in epithelial antiviral readiness. We find substantial variability in basal expression of PRRs such as RIG-I in uninfected ciliated cells, suggesting that only a subset of cells is poised to rapidly detect RSV and initiate IFN signaling. This heterogeneity may help explain conflicting reports on the relationship between IFN levels, viral load, and disease severity in RSV-infected patients (*47*, *58*). Our results suggest that the timing of IFN induction relative to viral replication may be a critical determinant of infection outcome, with early sensing favoring containment and delayed sensing enabling immune evasion.

Beyond immune suppression, RSV infection triggered a broad cellular stress response that intensified with increasing viral load. Genes associated with metabolic stress, unfolded protein response, and apoptosis were progressively induced, and the inhibition of apoptosis increased the number of detectable infected cells (fig. S4). These findings support a model in which RSV infection is balanced by continuous epithelial turnover: Infected ciliated cells are lost through stress-induced cell death, while basal cells differentiate to replenish the epithelium, maintaining a dynamic steady state of infection. In vivo, this balance is likely influenced by immune cell recruitment and regenerative capacity, which may be insufficient in immunocompromised patients, leading to persistent infection and severe disease (*59*, *60*).

Notably, not all antiviral regulators were suppressed during RSV infection. IRF1 remained stably expressed across the viral load gradient and exerted a potent antiviral effect when ectopically expressed, even under high viral inoculum conditions. Unlike exogenous IFN treatment, which delayed but did not prevent RSV replication, IRF1 overexpression conferred sustained protection and synergized with IFN signaling. IRF1 is known to regulate antiviral gene expression both dependently and independently of IFN signaling, to prime chromatin at antiviral loci, and to maintain expression of viral sensors (*61*, *62*). Our findings suggest that RSV is less effective at counteracting IRF1-mediated defenses, highlighting IRF1 as a promising candidate for host-directed antiviral strategies.

This study has several limitations. First, our experimental system lacks resident and infiltrating immune cells, preventing assessment of how epithelial reprogramming interacts with immune cell recruitment, cytokine networks, and adaptive immunity in vivo. Second, the use of adult donor tissue limits direct extrapolation to pediatric airways, where RSV disease burden is highest and epithelial and immune responses may differ. Third, functional validation of IRF1 was performed in a lung carcinoma cell line, and future studies will be needed to confirm its antiviral efficacy in primary airway epithelial cells and in vivo models. Last, while viral RNA load provides a useful proxy for infection progression, it does not capture all aspects of viral life cycle dynamics. Despite these limitations, our findings provide a unified framework for understanding how RSV reshapes the airway epithelium at single-cell resolution. By coupling viral load–dependent epithelial dedifferentiation with selective suppression of innate immune signaling, RSV creates a permissive niche for replication while avoiding widespread tissue destruction. Identifying antiviral regulators, such as IRF1, that escape viral antagonism offers a potential path toward host-directed therapies that reinforce epithelial resistance and complement existing prophylactic strategies. More broadly, our work highlights the value of viral load–resolved single-cell analysis for dissecting host-pathogen interactions in complex tissues. RSV dedifferentiates infected ciliated lung epithelial cells and suppresses antiviral immunity in a virus load–dependent manner.

## MATERIALS AND METHODS

### Ethic statement

Adult patients gave informed consent for the donation of lung samples, and all steps were in compliance with good clinical and ethical practice and approved by the local ethical committee at Hannover Medical School (permission number 3346/2016).

### Cell lines, ALI culture, and virus

A549 cells [American Type Culture Collection (ATCC); CCL-185] were cultured in F12K NutMix media supplemented with 10% heat-inactivated fetal calf serum (FCS) (Capricorn Scientific), 1% NEAA (Gibco), 2 mM l-glutamine (Gibco), penicillin (100 U/ml), and streptomycin (100 U/ml; Gibco) at 37°C and 5% CO_2_ in a humidified incubator. To generate cell lines stably expressing human IRF1 (hIRF1) or TXNIP, A549 cells were either transduced with lentiviral particles encoding the vector control (pWPI-BLR) or a lentiviral vector encoding for hIRF1 (pWPI-hIRF1-BLR) or human TXNIP (pWPI-TXNIP-BLR), respectively. Three days posttransduction, cells were selected for efficiently transduced cells by addition of blasticidin as selection antibiotic.

Primary human airway epithelial cells cultured under air-liquid-interface (ALI) conditions were cultivated and differentiated as described elsewhere (*63*). In brief, human airway epithelial cells were isolated from the bronchus of explanted human lungs by enzymatic digestion for 48 hours at 4°C. Cells were seeded on collagen I/III-coated flasks for proliferation before seeding on collagen IV–coated transwells (0.4-μm pore size, polyester membrane inserts, Corning Costar). All cells were routinely tested negative for *Mycoplasma* contamination (Eurofins MWG).

The recombinant human respiratory syncytial virus subtype A GFP (rHRSV-A-GFP) reporter virus was described elsewhere (*40*). Virus stocks were prepared in HEp-2 cells (ATCC, CCL-23). For harvesting, infected cells were scraped and vigorously vortexed to release cell-bound virus particles. Virus preparation was centrifuged for 10 min at 1000*g* to remove cell debris, supplemented with virus stabilizer [final concentration of 100 mM MgSO_4_ and 50 mM Hepes (pH 7.5)], aliquoted, and snap frozen in liquid nitrogen. Virus titer was determined using the limiting dilution assay (TCID_50_).

### Cell preparation and library preparation for single-cell RNA-seq

Differentiated primary human airway epithelial cells from six different donors were inoculated with recombinant RSV-A–GFP reporter virus for 2 hours at 37°C (1.4 × 10^5^ infectious virus particles per transwell) or with conditioned media generated in parallel to the virus stock. After 2 hours, the viral inoculum was removed and the apical compartment was washed twice with Hank’s balanced salt solution (HBSS). At different timepoints post virus inoculation, cells were washed apically with HBSS before cell detachment by addition of trypsin/EDTA and incubation at 37°C. Then, cells from the different donors belonging to the same time point were pooled before hashtagging (TotalSeq-A Antibodies, BioLegend). RSV-infected (GFP-positive) cells were sorted using a FACS Aria III and 3000 GFP-positive and 6000 bystander cells per time point were loaded in the 10x Genomics controller. In parallel and simultaneously, noninfected cells were trypsinized, hash-tagged for the time point, and applied for the 10x Genomics (see below).

Cell suspensions at a density of 400 cells/μl in phosphate-buffered saline (PBS) + 0.04% bovine serum albumin (BSA) were prepared for single-cell sequencing. Chromium Controller was used for partitioning single cells into nanoliter-scale gel bead-in-emulsions (GEMs). Briefly, 5 to 10,000 cells per reaction were loaded for GEM generation and barcoding. Libraries were constructed using the Chromium Single Cell 3′ Reagent Kit v2 and v3 (10x Genomics), following the manufacturer’s instructions. A SimpliAmp Thermal Cycler was used for amplification and incubation steps (Applied Biosystems). Libraries were quantified by a QubitTM 3.0 fluorometer (Thermo Fisher Scientific), and the quality was checked using a 2100 Bioanalyser with a High Sensitivity DNA kit (Agilent Technologies). Samples were pooled and sequenced using the Illumina NovaSeq 6000 in a paired-end mode: read 1, 26 cycles; index 1, 8 cycles; index 2, 0 cycles; and read 2, 98 cycles. 

### Live-cell imaging

Cells were seeded into 96-well plates at 7 × 10^3^ cells per well in a total of 100 μl of F12K NutMix cplt media. Eight hours postinfection, cells were treated with indicated concentrations of human IFN alpha-2b (IntronA, MSD Sharp&Dome GmbH) where indicated. Sixteen hours post-IFN treatment, cells were inoculated with an RSV-A–GFP reporter virus at indicated multiplicity of infection for 3 hours in a total of 50 μl. After inoculation, viral inoculum was removed and replaced by 100-μl media supplemented with IFN as indicated. The scanning of cells started 4 hours postinoculation using an Incucyte SX5 machine (Sartorius BioAnalytical Instruments). Four pictures each from at least two wells were scanned every 4 hours using a ×10 magnification. Total green integrated intensity (GCU × μm^2^ per image) was accessed using the Incucyte software (GUI Version 2023A, controller version 2023A Rev1, firmware version 20231.1.4.0 RTM).

### Flow cytometry analysis (A549 cells)

Cells were seeded into 96-well plates at 1 × 10^4^ cells per well the day before infection with the recombinant reporter virus RSV-A–GFP at different multiplicities of infection for 4 hours. Twenty-four or 48 hours postinoculation, cells were trypsinized and fixed in 3% paraformaldehyde in PBS for at least 30 min. Cells were stored at 4°C upon flow cytometric analysis using a SONY spectral analyzer SA3800, and results were analyzed using FlowJo V10. Gating strategy is depicted in fig. S1C.

### Flow cytometry analysis of well-differentiated primary human airway epithelial cells

Well-differentiated primary human airway epithelial cells from three donors were inoculated with recombinant reporter virus RSV-A–GFP (4 × 10^5^ focus-forming units per well) or treated with conditioned media for 2 hours before the removal of inoculum. Cells were cultured at 37°C and 5% CO_2_. At the indicated time points, cells were detached using Accutase (BioLegend, reference no. 423201) and fixed with 3% paraformaldehyde/PBS. Fixed cells were permeabilized for 30 min on ice in 0.1% saponin-containing 0.5% FCS/PBS before incubation with primary antibody for 1.5 hours at 4°C. After multiple washing steps with 0.5% FCS/PBS, cells were incubated with the respective Alexa Fluor 647–labeled secondary antibody for 1 hour on ice before extensive washing and analysis of cells using a SONY spectral analyzer SA3800 and FlowJo V10 software. Gating was performed using the respective isotype control antibodies on living, single cells (fig. S6, A to C). The percentage of positive cells were corrected for the background using the isotype control antibody-stained cell population.

The following primary antibodies were used: anti-CD49f (Invitrogen, #14-0495-85), anti-ARL13B (Proteintech, #17711-1-AP), phycoerythrin (PE)–anti-CD271 (BioLegend, #345106), anti-Mucin5AC (Novus, NBP2-15196), anti–ß-tubulin–Cy3 (Sigma-Aldrich, C4582), anti-rat IgG kappa (Invitrogen, #14-4321-81), anti-rabbit IgG (Abcam, ab37415), anti-mouse IgG1 kappa (BD Biosciences, #555746), and anti-mouse Cy3-IgG1 kappa (Bioss antibodies, #bs-0330R-Cy3). The following secondary antibodies were: Alexa Fluor (AF) 647 anti-rat (Invitrogen, A21247), AF647 anti-mouse (Invitrogen, A21235), and AF647 anti-rabbit (Invitrogen, A21244).

### Immunofluorescence analysis

RSV-A–GFP–infected differentiated primary human airway epithelial cells grown under ALI conditions were fixed with 4% formalin solution for at least 30 min at room temperature from the apical and basolateral side. After the removal of the fixation solution, both compartments were washed three times with PBS before addition of the blocking buffer [50 mM NH_4_Cl, 0.1% saponin, and 2% BSA in PBS (pH 7.4)] for 1 hour. Blocking buffer was replaced by antibody dilutions to the apical compartment and incubated for 2 hours at room temperature. After binding of first antibody, the apical surface was washed three times with blocking buffer before addition of the conjugated secondary antibodies to the apical compartment for 1 hour. Unbound antibodies were washed away by 3× PBS washes, the membrane was excised from the transwell and mounted on glass cover slides. Immunofluorescence acquisition and analysis were performed using an Olympus microscope (FV3000 and CellSens software, Olympus).

The following primary antibodies were used (*64*): anti-CD49f (Invitrogen, #14-0495-82), anti–β-tubulin–Cy3 (Sigma-Aldrich, C4585), anti-cytokeratin 5 (Abcam, ab52635), anti-ARL13B (Proteintech, #14411-1-AP), anti-Mucin5AC (Santa Cruz Biotechnology, sc-21701), and anti–CD271-PE (BioLegend, #345106). The following secondary antibodies were: AF647 anti-rat (Invitrogen, A21247), AF647 anti-rabbit (Invitrogen, A21244), AF568 anti-rabbit (Invitrogen, A10042), and AF647 anti-mouse (Invitrogen, A21235).

### scRNA-seq data processing, quality control, cell-type annotation, and general data analysis

The fastq-files were mapped using the cellranger pipeline (version 7.0.0). The human genome sequence was taken from the ENSEMBL database (version 90), and the RSV sequence was taken from Genbank (accession number MK816924). Data analysis was performed in R (version 4.3). Filtering, normalization, feature selection, and dimensionality reduction were performed using the Seurat package (version 4.3.0). Visualization was done using the ggplot2 package (v. 3.5.1).

At first, time points were demultiplexed using the Seurat package. First, the hashtag read counts were normalized using NormalizeData with the centered log ratio transformation as normalization method and then, the HTODemux function was used with positive.quantile = 0.998. Subsequently, all hashtag negative (*n* = 7238) and doublet cells (*n* = 849) were removed.

We kept all features that were detected in more than three cells and filtered cells according to the following statistics: Number of features (1500 < × < 10,000), number of UMIs (5000 < × < 100,000), percentage of mitochondrial reads (<30%), and number of viral UMIs (<50 in bystander cells, >100 in infected cells; see Fig. 1B and fig. S2A).

To annotate the cell types, we used the IntegrateData function of the Seurat package (v. 4.3.0) with standard parameters to integrate all mock cells of our data into a single-cell atlas of the human lung (*41*) and calculated the nearest neighbor graph (*k* = 50) on the integrated dataset. Subsequently, we iterated over all unannotated mock cells and labeled each cell according to the most prevalent cell type in its nearest neighborhood. After mock cell annotation, we integrated the mock and unannotated virus-exposed cells using standard parameters, calculated the nearest neighbor graph (*k* = 20), and labeled the virus-exposed cells according to their neighborhood to the mock cells as described above.

We removed all cell types with less than 10 cells and further scrutinized all remaining cells after clustering. This led us to remove another set of cells that survived the general filtering thresholds defined above but had more than twice the number of mitochondrial reads and an order of magnitude less detected UMIs than all other cells (see fig. S1, B to D).

Dimensionality reduction was performed on the integrated assay using the RunPCA function with standard parameters and the RunUMAP function with 35 dimensions from Seurat. The viral RNA load of cells was calculated as the log of total sum of normalized viral gene counts per cell.

The donor cells were demultiplexed by genetic variants using souporcell (v. 2.0) from a singularity container (v. 3.9.1+84-g510833307) with the following command: “singularity exec souporcell.sif souporcell_pipeline.py -i bamfile.bam -b barcodes.tsv -f reference.fasta” with the bam-file and barcodes from the Cell Ranger output and the human genome sequence from the ENSEMBL database (version 90). We discarded all cells that were not assigned as singlets.

### Viral gradient plot and manual RSV annotation

For the viral gradient plot (Fig. 1E), we inspected read coverage in the RSV genome in a genome browser (display function of the GEDI toolkit version 1.0.6). We created a manual annotation of the 11 poly(A) sites in the RSV genome by extending the visually identified read count peaks identified in the genome browser by 200 bp on both sides. We then reran the Cell Ranger pipeline with the updated annotation and then determined the proportion of raw read counts for each viral gene relative to the total viral read counts per cell and plotted them in order based on their position in the viral genome. The manual annotation of the RSV genome for 3′ end peaks is available as data S1.

### GO analysis

GO analysis was performed in R using the clusterProfiler package (version 4.8.1) using all genes in the respective cluster as the targets, all genes in the dataset as the background, and the org.Hs.eg.db package (version 3.17.0) as the database. We adjusted *P* values using Benjamini-Hochberg correction and set a *q* value threshold of 0.05 for all GO analyses and a log fold change (LFC) threshold of −0.5 for the GO analysis of down-regulated genes in basal-like compared to ciliated cells (Fig. 3D).

### Infection time analysis of infected cells

For the infection time analysis we only included cells annotated as ciliated, proximal ciliated, proximal basal, and basal cells. We sorted the infected cells by the number of viral reads and binned them into 20 pseudobulks each consisting of *n* = 85 to 86 cells, pooling reads of all cells per pseudobulk. Similarly, we pooled the reads of all mock and bystanders into a pseudobulk, respectively. We then calculated normalized LFCs versus mock cells using the PsiLFC function of the lfc package (version 0.2.3) with standard parameters (*65*). Differential gene expression analysis between pseudobulks of infected cells on the 5000 most variable genes was performed using the DESeq2 package (version 1.40.1). We included all genes with an adjusted *P* value < 0.01 (*n* = 1033) in the analysis.

To cluster genes by their progression over infection time rather than absolute LFC values, we centered each gene by subtracting its mean LFC from all LFCs of that gene. A Seurat object was created from the LFC values of the bystander and infected pseudobulks, loading genes as observations and pseudobulks as features. The data was then scaled and principal components analysis (PCA) was conducted using the ScaleData and RunPCA functions with standard parameters, and the nearest neighbor matrix (using the FindNeighbors function and the first 20 dimensions) was calculated. Last, clustering was performed using the FindClusters function with a resolution of 1.5. The gene-based UMAP was then constructed with the RunUMAP function on the first 20 principal components. In all cases, euclidean distance was used.

The line plots (Fig. 7A) show the smoothed LFCs versus mock of all genes in all pseudobulks per cluster and a regression line over all genes per cluster. The lines were created using the geom_smooth function of ggplot2 with “lm” as the smoothing function and the formula *y* ~ splines::bs(*x*, 3). To improve the visualization and remove outliers, the *y* axis was limited to LFCs in [−4,4].

### Statistical analyses

Statistical analyses were conducted using GraphPad prism software (version 10.0.2) and are described in the figure legends. Error bars and replicate numbers are defined in the respective figure legends.
