# Supplementary material for: Respiratory syncytial viral load drives ciliated cell dedifferentiation and suppresses antiviral immunity
Source: Sci Adv. 2026 Jun 19;12(25):eaed4499. doi: 10.1126/sciadv.aed4499 (PMC13281798; doi:10.1126/sciadv.aed4499)
Supplement: Supplementary file 1 — Figs. S1 to S12 Legend for table S1 Legend for data S1 [file sciadv.aed4499_sm.pdf]

Science Advances
AAAS

Supplementary Materials for

Respiratory syncytial viral load drives ciliated cell dedifferentiation and suppresses antiviral immunity

Kevin Berg *et al.*

Corresponding author: Antoine-Emmanuel Saliba, emmanuel.saliba@helmholtz-hiri.de;
Florian Erhard, florian.erhard@informatik.uni-regensburg.de; Thomas Pietschmann, thomas.pietschmann@twincore.de

Sci. Adv. **12**, eaed4499 (2026)
DOI: 10.1126/sciadv.aed4499

The PDF file includes:

Figs. S1 to S12
Legend for table S1
Legend for data S1

Other Supplementary Material for this manuscript includes the following:

Table S1
Data S1

Supplementary Figures

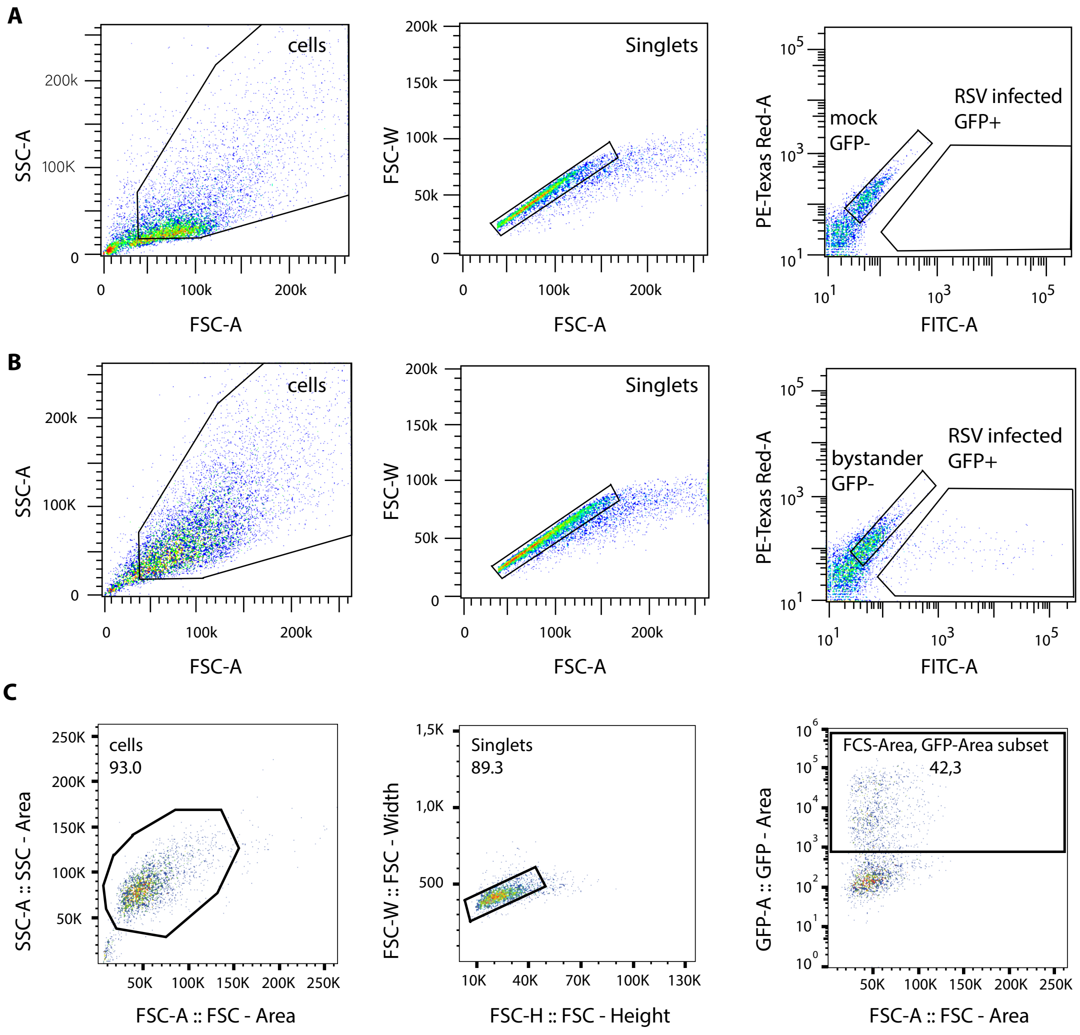

Supplementary Figure S1: Fluorescence-activated cell sorting strategy for RSV-infected and bystander cells. ***(A)*** *Uninfected and* ***(B)*** *RSV-GFP infected primary human airway epithelial cells 3 days post inoculation were FACS sorted using a BD FACSDiva system. Cells were gated for living and singlet cells prior to sorting for GFP-negative bystander cells and GFP-positive RSV-infected cells.* ***(C)*** *Gating strategy for A549 cell lines infected with RSV-A-GFP (MOI1) for 24h.*

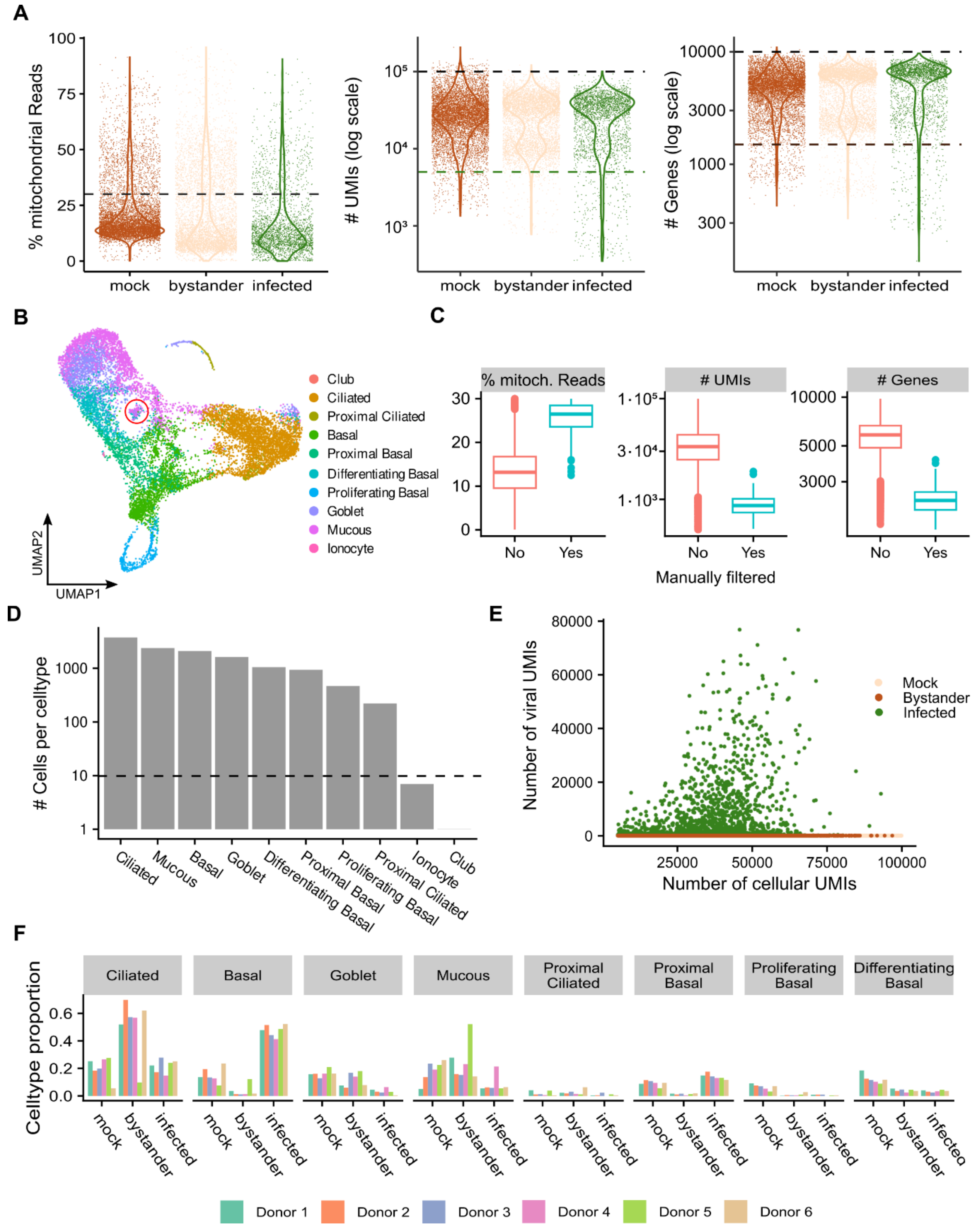

Supplementary Figure S2: Quality control and preprocessing of single-cell RNA-sequencing data. (***A)*** *Violin plots of the percentage of mitochondrial reads (left), number of detected unique molecular identifier (UMIs) (middle) and number of detected genes (right) per cell, split by infection state. Dashed lines indicate the filtering cut-offs.* ***(B)*** *UMAP representation as in Figure 1D of annotated cells that passed basic quality control measures (12537 cells). Ionocyte (7 cells) and club cells (1 cell) as well as cells with a combination of very high mitochondrial reads and low read and feature counts (198 cells, red circle) were removed from the analysis.* ***(C)*** *Boxplots of the percentage of mitochondrial reads, number of detected UMIs and number of detected genes per cell for the manually removed cells (red circle in* ***(A)****) and the remaining data set.* ***(D)*** *UMAP representation of single-cell RNA-seq annotated and color-coded according to lung atlas-based cell type annotation.* ***(E)*** *Scatter plot showing the number of cellular UMIs versus the number of viral UMIs in mock (beige), bystander (brown) and infected (green) cells.* ***(F)*** *Relative proportion of cell profiles per infection state decomposed according the donor of origin.*

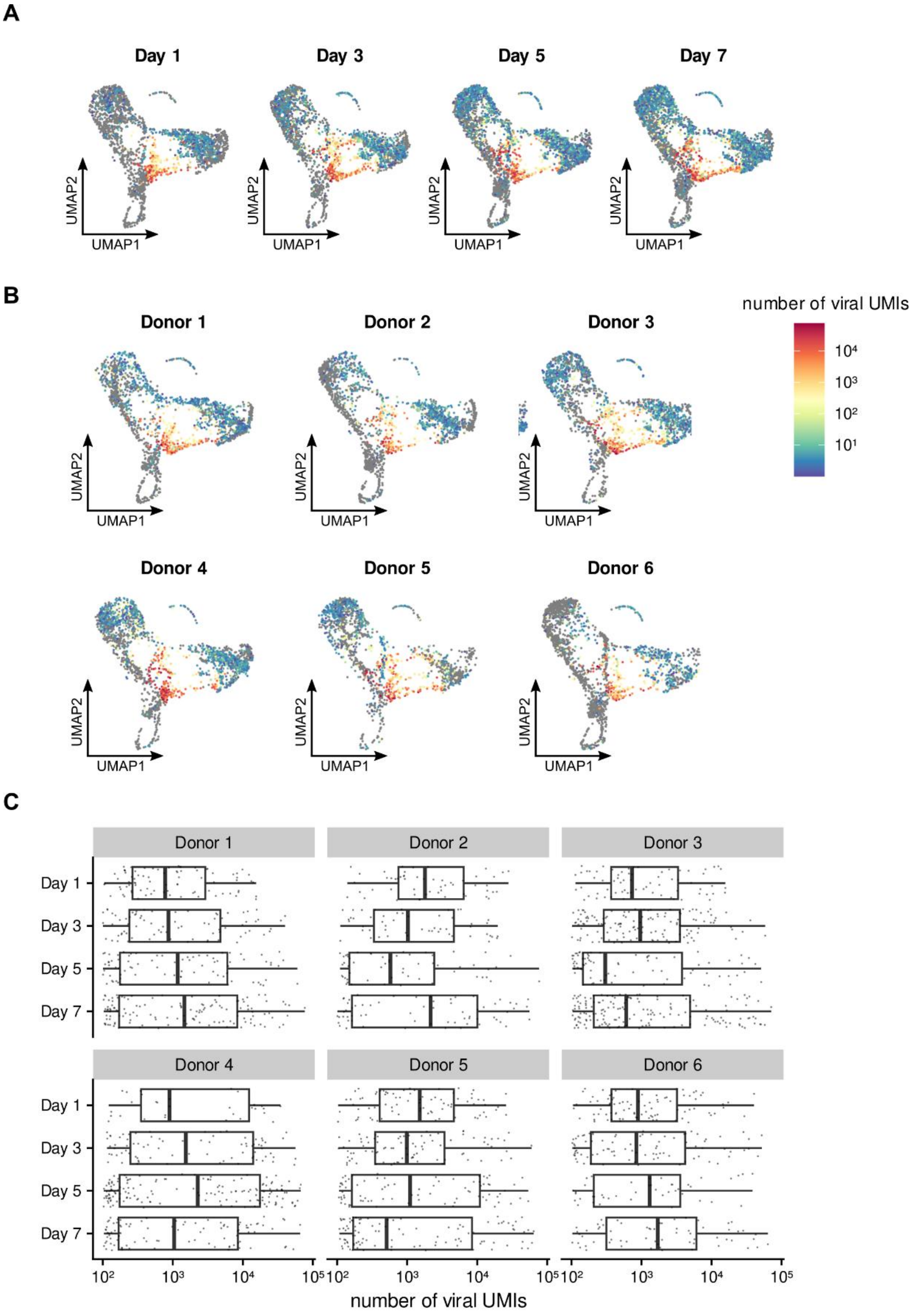

Supplementary Figure S3: Distribution of viral RNA loads across time points and donors. *(**A**) UMAP representation of viral UMIs in infected cells across all time points. (**B**) UMAP representation of viral UMIs in infected cells across all donors. (**C**) Boxplots showing the number of viral UMIs in infected cells across all time points and donors.*

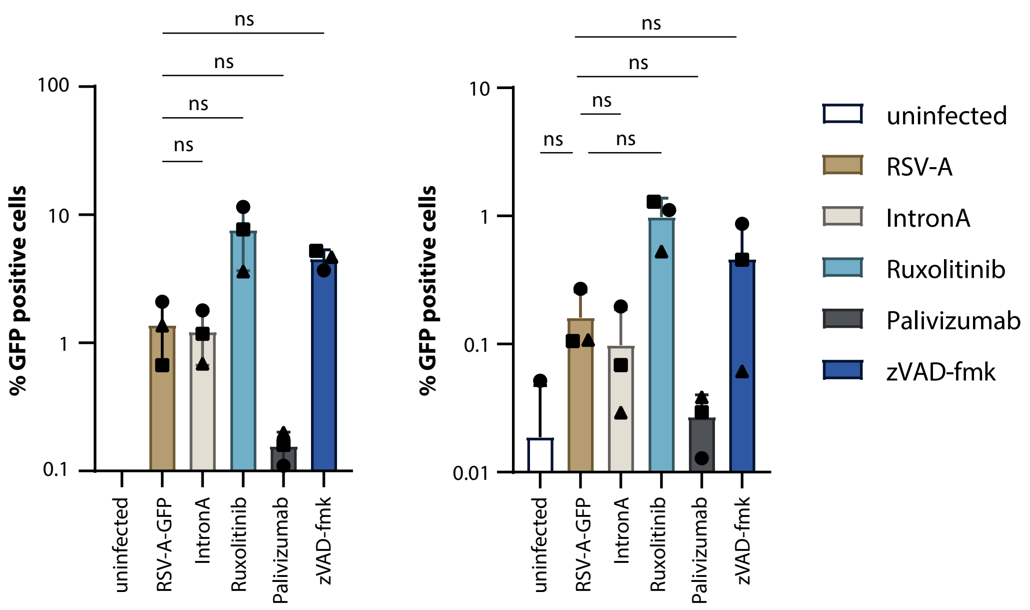

Supplementary Figure S4: Effect of pharmacological modulation of antiviral and apoptotic pathways on RSV infection. *Well-differentiated primary human airway epithelial cells were inoculated for one hour with an RSV-A-GFP reporter virus. 24h post inoculation, cells were treated with 1000 IU/mL IntronA, 10 µM Ruxolitinib, 10 µg/mL Synagis or 10 µM zVAD-fmk from both sides. To maintain air-liquid interface cultures, the drugs from the apical compartment were removed after 1h and apical treatment was repeated daily while basolateral treatment continued throughout the experiment. 120h post infection, cells were trypsinized and analyzed by flow cytometry using a SONY spectral analyzer SA3800. Experiments were performed twice, each with 3 independent lung cell donors.*

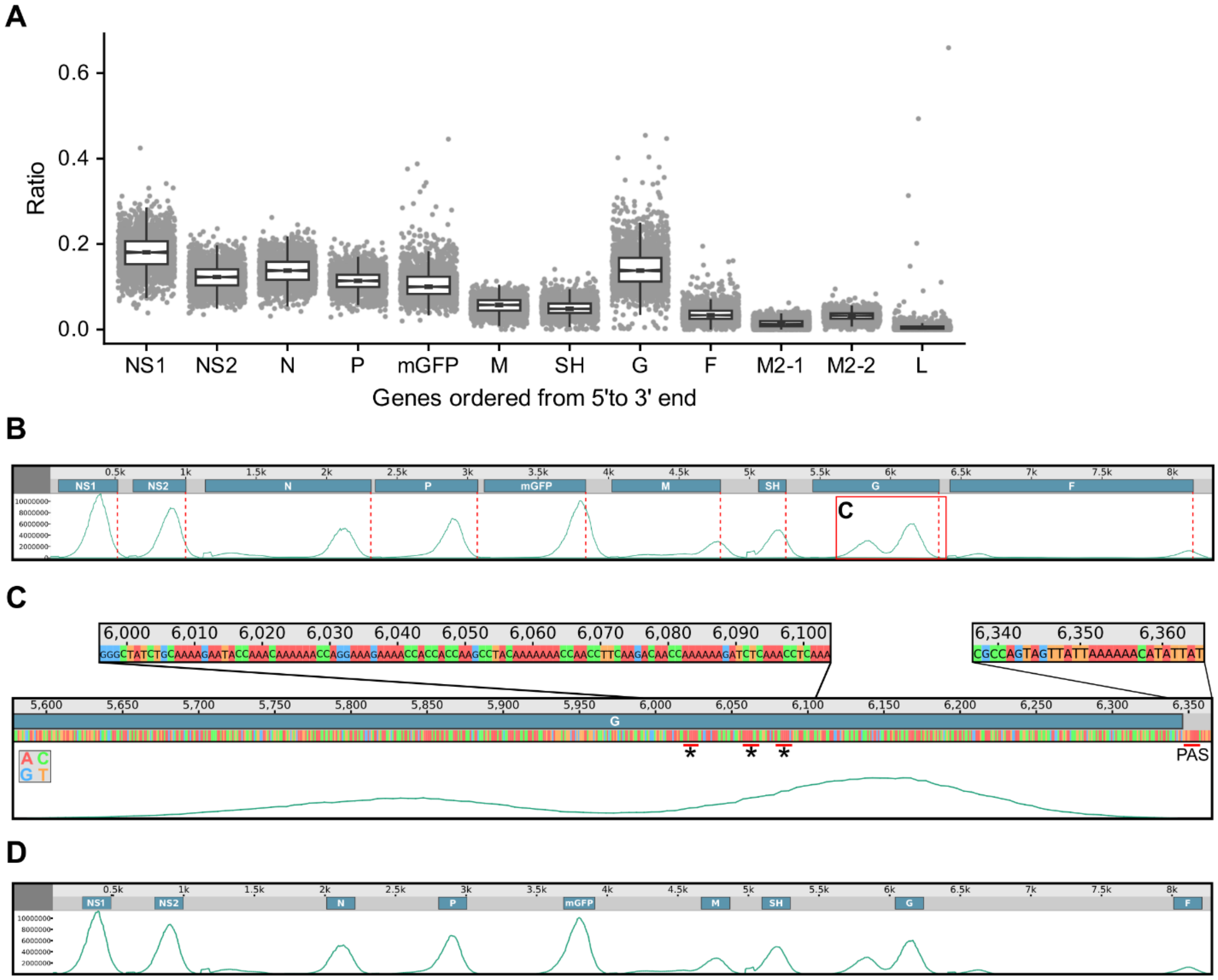

Supplementary Figure S5: RSV genome coverage and manual reannotation for transcriptional gradient analysis. ***(A)*** *Dot plot of fraction of reads mapped to viral mRNAs with superimposed box plot (XdescriptionX) before manual RSV reannotation.* ***(B)*** *Genome Viewer of the RSV genome annotation. Dashed red lines mark the end of annotated genes. Red box labeled ‘C’ of G segment indicates the part of the genome analysed in panel C in detail.* ***(C)*** *Genome viewer magnified on the G segment with the polyA signal and several polyA-like signal sequences are highlighted by an asterisk and magnified.* ***(D)*** *Genome viewer of the manual RSV genome annotation covering 200 bp windows centered on the 3’ read count peaks.*

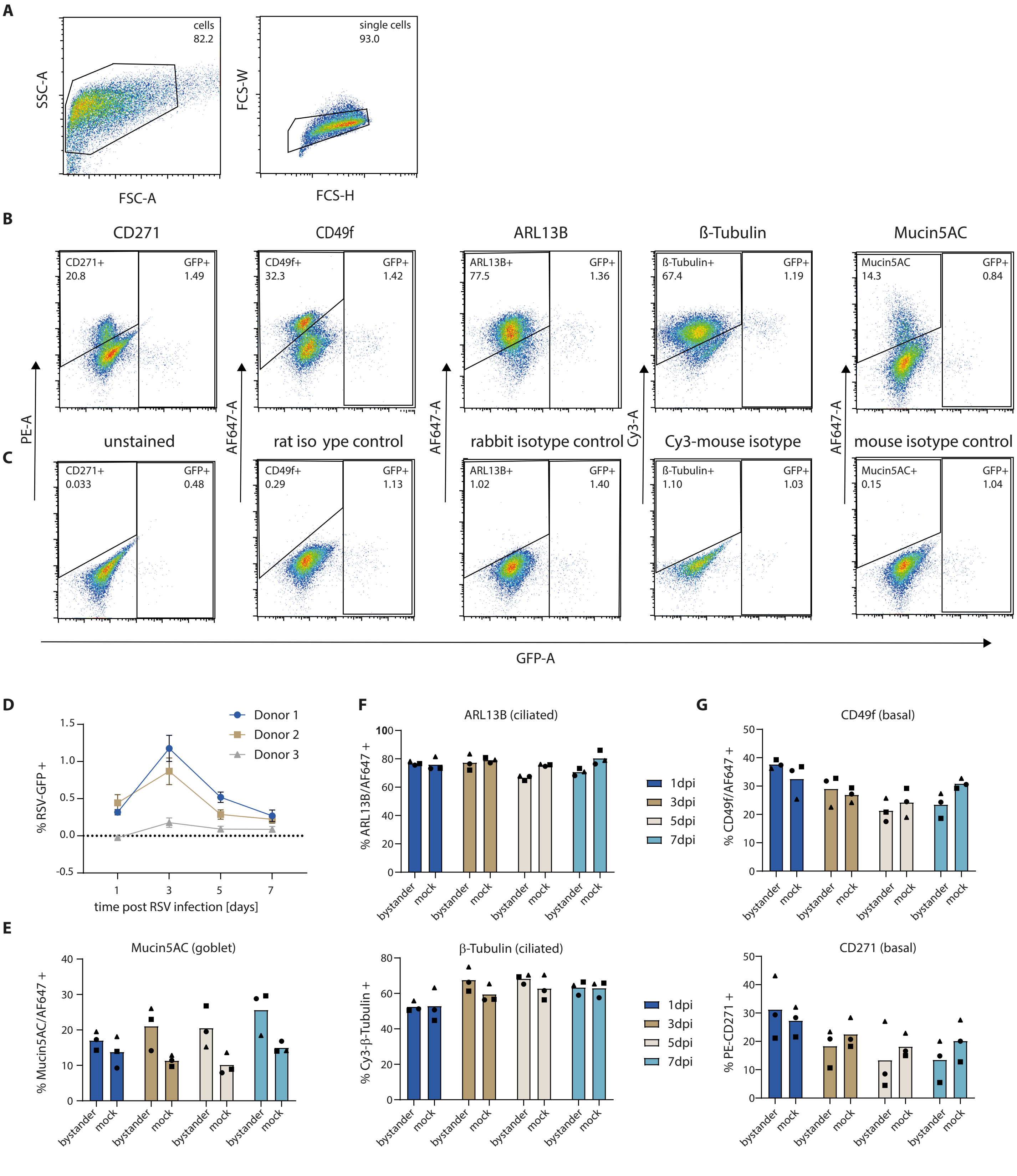

Supplementary Figure S6: Expression of marker genes using flow cytometry analysis. (**A**) *Gating strategy for living and single cells.* (**B**) *Gating strategy for RSV-infected, GFP-positive cells, marker gene expression and* (**C**) *corresponding isotype control. One representative donor is given.* (**D**) *Percentage of GFP-positive cells over time from n=3 independent donors. Percentage of cells positive for* (**E**) *Mucin 5AC,* (**F**) *ciliated markers ARL13B and β-Tubulin and* (**G**) *basal markers CD49f and CD271 are given. Bars represent the mean percentage of all 3 donors, symbols give the results from each donor separately.*

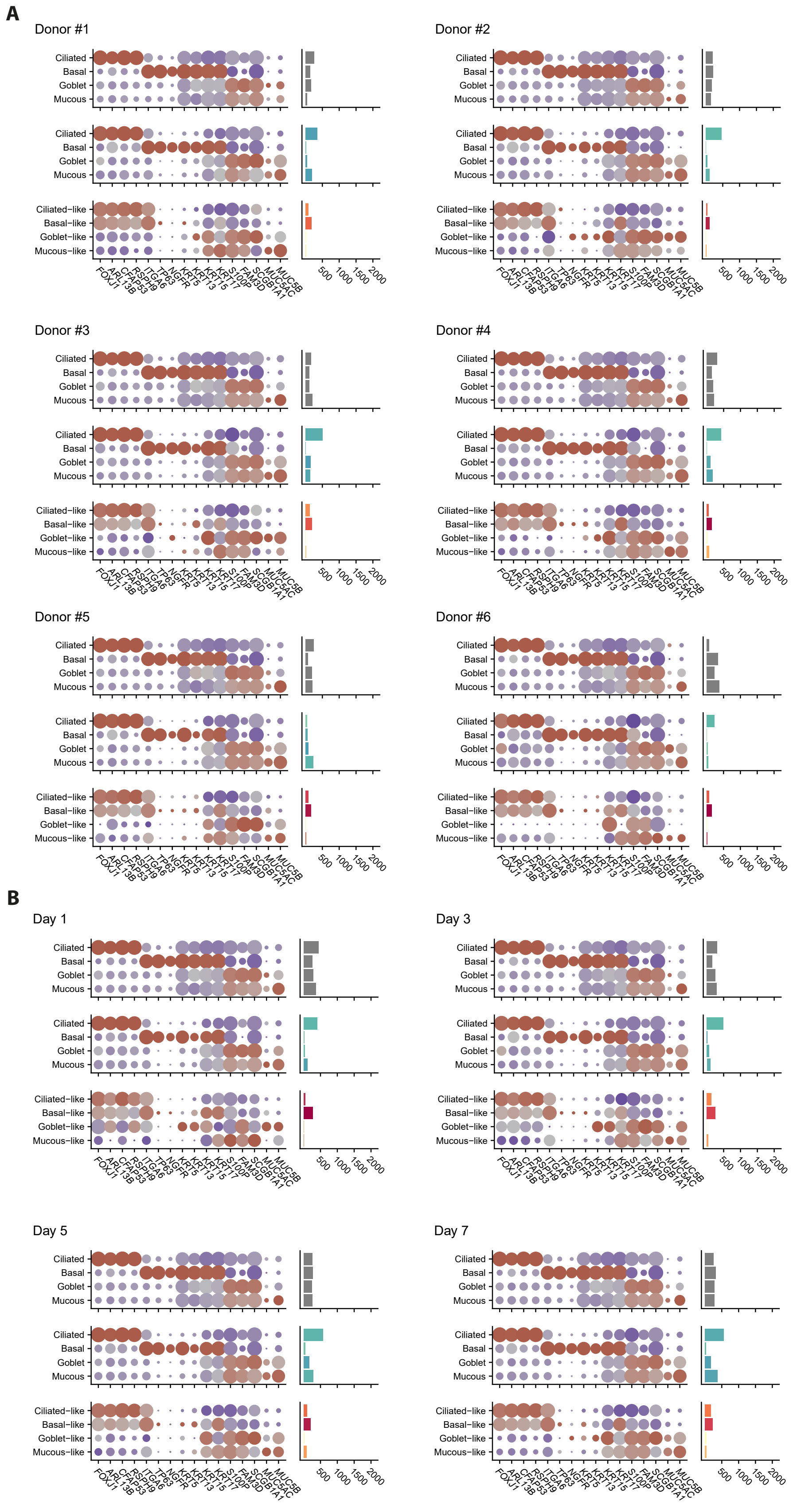

Supplementary Figure S7: Donor- and time dependent differentiation changes induced by RSV. (**A**) *Donor-dependent expression and percentage of expressing population of marker genes in mock, bystander and infected ciliated, basal, goblet and mucous cells (left). Cell counts per cell type and infection state, the color represents the average natural log value of viral reads (right).* (**B**) *Infection time-dependent expression and percentage of expressing population of marker genes in mock, bystander and infected ciliated, basal, goblet and mucous cells (left). Cell counts per cell type and infection state, the color represents the average natural log value of viral reads (right).*

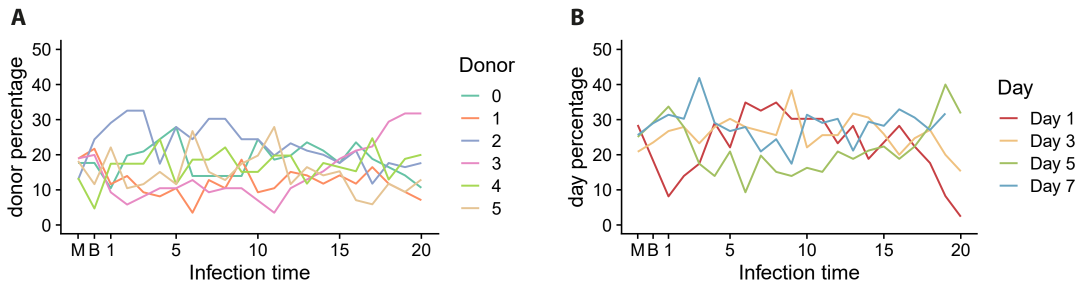

Supplementary Figure S8: Pseudobulk bins along infection timeline. *(**A**) Donor-dependent and (**B**) infection-time dependent pseudobulk bins along the infection time.*

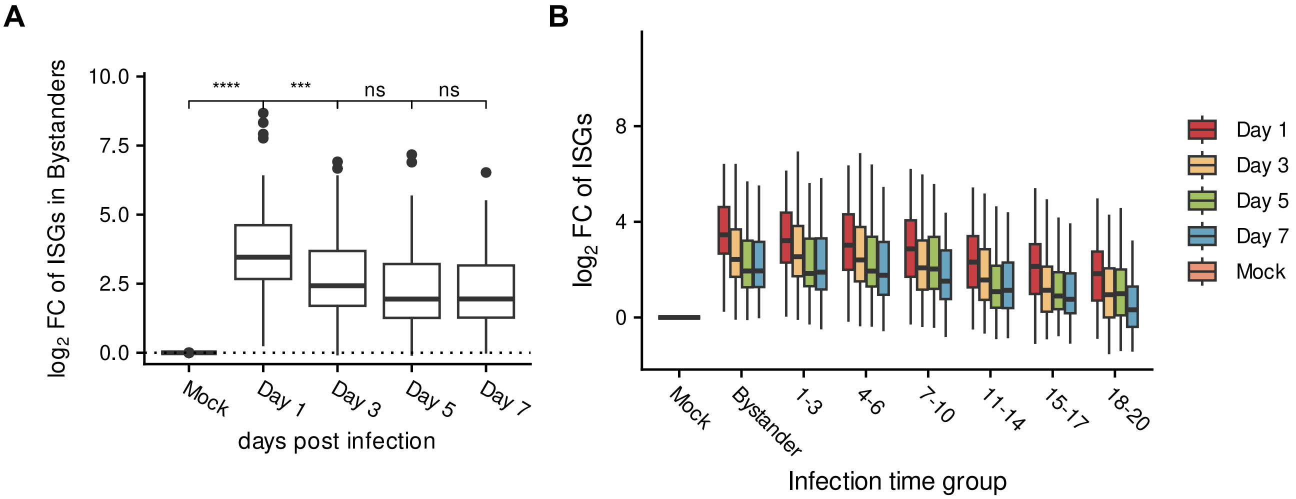

Supplementary Figure S9: Temporal dynamics of interferon-stimulated gene expression. ***(A)*** *Boxplots showing the log2 fold changes of ISGs across all time points versus all mock cells.* ***(B)*** *Boxplots showing the log2 fold changes of bystander cells and infection time groups and separated by time points.*

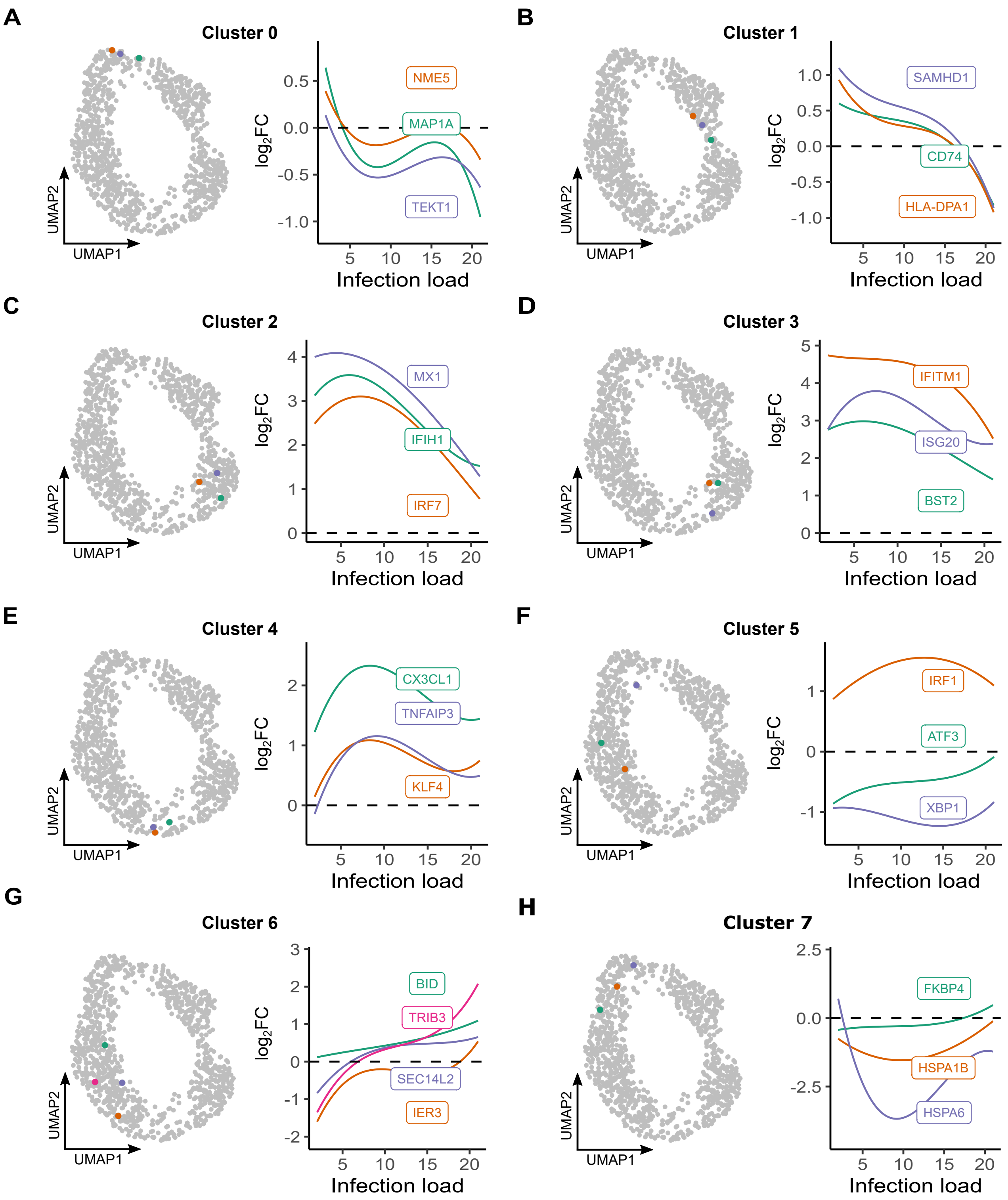

Supplementary Figure S10: Visualization of representative genes across viral load–dependent transcriptional clusters. ***(A-H, left)*** *UMAP representation of all differentially expressed genes over the Infection time, based on their* log_2FC *values in all pseudobulks. Exemplary genes for every cluster are highlighted.* ***(A-H, right)*** *Line plots of mean* log_2FC *values versus Mock over infection time for exemplary genes per cluster.*

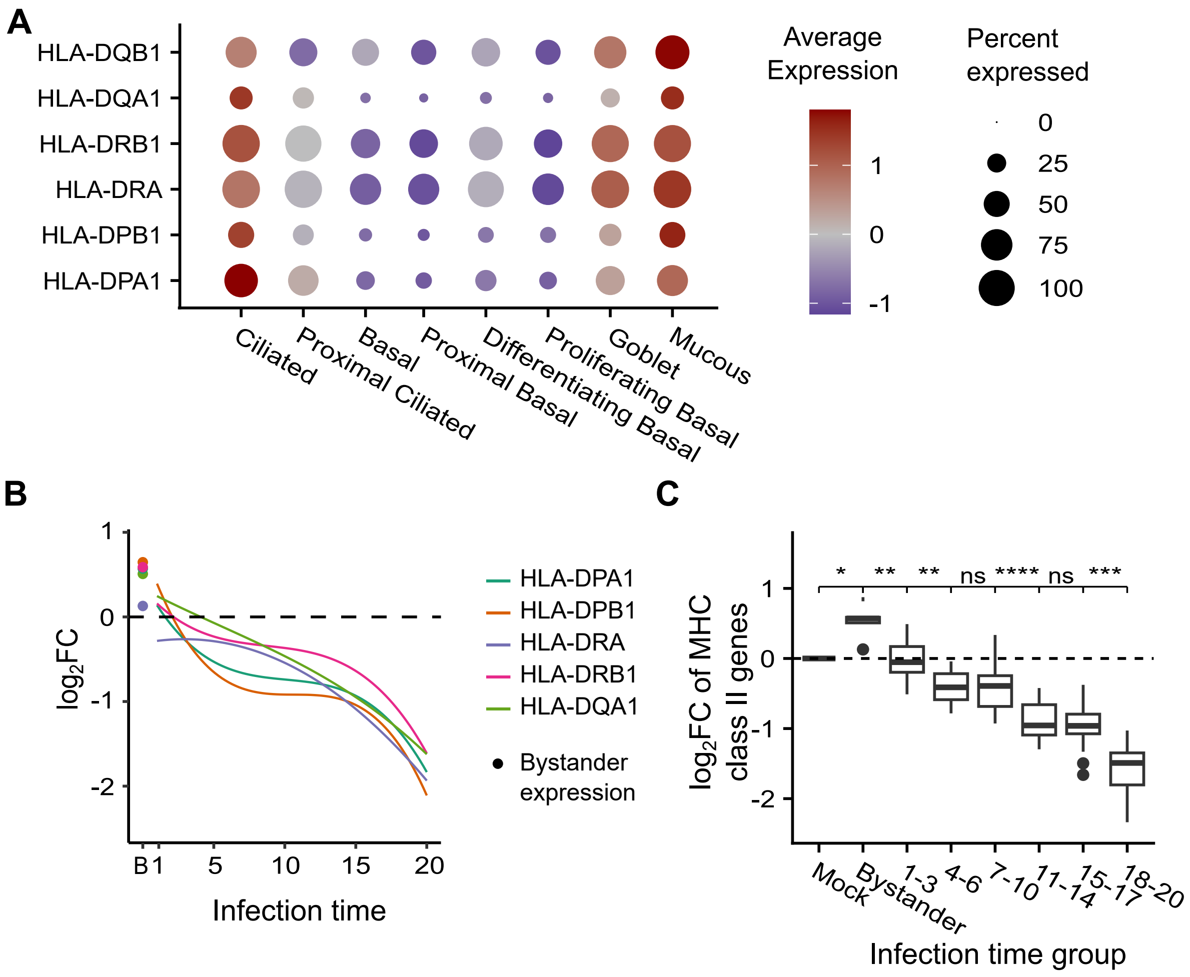

Supplementary Figure S11: Regulation of MHC class II gene expression during RSV infection. ***(A)*** *Average expression and percentage of expressing population of MHC class II genes in mock over all cell types.* ***(B)*** *Mean log_2FC values of differentially expressed genes versus Mock in bystander and infected cells over infection time. HLA-DQB1 was not differentially expressed.* ***(C)*** *Boxplots of the mean log_2FC of differentially expressed MHC class II genes in mock, bystander and infected cells over Infection load. Wilcoxon test, p-values indicated.*

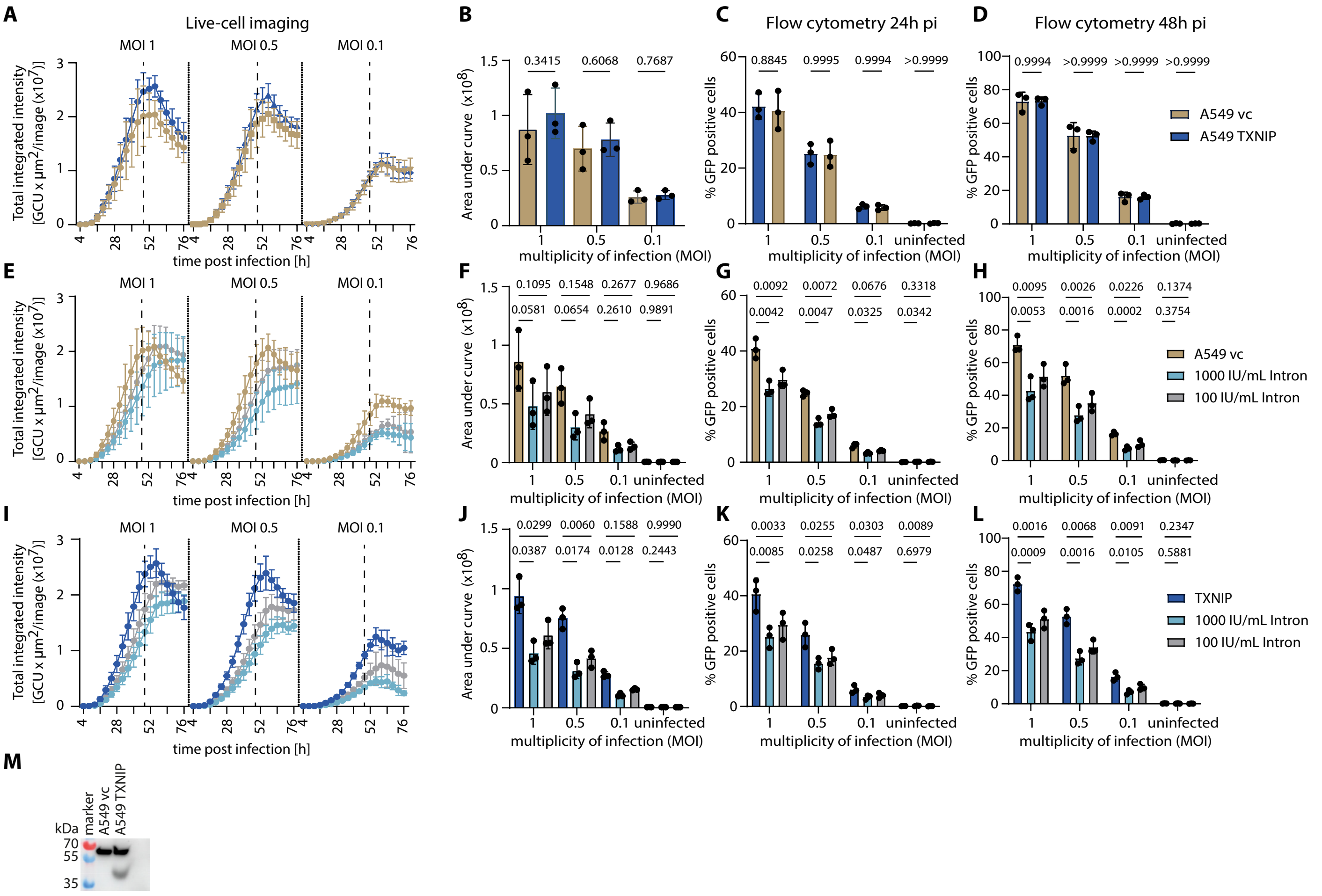

Supplementary Figure S12: TXNIP overexpression does not restrict RSV replication. *(**A**) A549 vector control (brown) or A549 cells stably overexpressing TXNIP (blue) were inoculated with an RSV-A-GFP reporter virus at indicated multiplicities of infection (MOI) and GFP expression was quantified by live-cell imaging. (**B**) Area under the curve from (**A**) until 48h pi was calculated and the statistical analysis was performed using a repeated-measurement one-way ANOVA in combination with Sídák´s multiple comparison test. Mean and std dev of n=4 independent experiments (**A+B**) as well as the results from the single experiments (symbols, **B**) are given. (**C, D**) Flow cytometric analysis at 24h (**C**) and 48h (**D**) post infection. Bars represent mean and std dev. of n=3 independent experiments. Two-way ANOVA with Sídák´s multiple comparison test. (**E-H**) RSV-GFP infection data of given cells pretreated with given quantities of interferon-2 alpha. (**E, F**) Live-cell imaging and statistical analysis of n=3-4 independent experiments. (**G, H**) Flow cytometric analysis 24h pi (**G**) and 48 pi (**H**) of n=3 independent experiments. Bars represent mean and standard deviation including the results for each independent experiment (symbol). Two-way ANOVA with Sídák´s multiple comparison test. (**I-L**) A549 TXNIP cells pretreated with interferon-2 alpha and RSV infected. (**I, J**) Live-cell imaging analysis (n=3 independent experiments) using two-way ANOVA with Sídáks multiple comparison test. (**K, L**) Flow cytometric analysis 24h pi (**K**) and 48 pi (**L**). Bars represent mean and std dev. of n=3 independent experiments. Two-way ANOVA with Sídák´s multiple comparison test. (**M**) TXNIP protein expression of vector control cells or cells stably transduced with TXNIP.*

Supplementary Table S1

Gene ontology enrichment analysis of viral load–dependent transcriptional clusters.

Supplementary Data 1

Manual RSV genome annotation used for transcriptional gradient analysis.
Gene transfer format (GTF) file defining 200 bp windows centered on 3′ viral read peaks.
